# SApneaNet: Adaptive Squeeze-and-Excitation-Based CNN–Transformer Network with AGFF for Sleep Apnea Event Detection Using ECG Images Under IoMT

**DOI:** 10.3390/s26185936

**Published:** 2026-09-19

**Authors:** Innocent Tujyinama, Bessam Abdulrazak, Rachid Hedjam

**Affiliations:** 1 Ambient Intelligence Lab, Department of Computer Science, Faculty of Science, University of Sherbrooke, Sherbrooke, QC J1K 2R1, Canada; bessam.abdulrazak@usherbrooke.ca (B.A.); rhedjam@ubishops.ca (R.H.); 2 Department of Computer Science, Bishop’s University, Sherbrooke, QC J1M 1Z7, Canada

**Keywords:** electrocardiogram, continuous wavelet transform, RGB log-scalogram, deep learning, adaptive squeeze-and-excitation, adaptive gated feature fusion, obstructive sleep apnea detection, IoMT

## Abstract

**Background and Objective:** Obstructive sleep apnea (OSA) is a fatal widespread sleep-related breathing disorder and a major risk factor for cardiovascular and cerebrovascular diseases, significantly impacting older adults’ health worldwide. Due to the risk of such complications, timely and accurate identification of OSA is crucial. Polysomnography is considered the most accurate technique for detecting OSA; however, it is limited by its complexity and multi-channel requirements. A promising alternative is electrocardiogram (ECG)-based diagnosis, which continuously monitors heart rhythm and captures subtle cardiac changes associated with OSA. Nevertheless, existing ECG-based approaches still face challenges related to complex feature engineering, limited capture of complementary temporal–spectral information and global dependencies, along with inadequate feature recalibration and fusion, which can restrict OSA detection. Thus, further improvements are still required to achieve clinically reliable performance. **Methods:** To address these challenges, this study proposes SApneaNet, a novel advanced deep learning method for detecting OSA events using ECG signals. The proposed approach employs the continuous wavelet transform (CWT) to convert ECG signals into RGB log-scalograms, enabling the simultaneous analysis of temporal and frequency-domain features. The generated RGB log-scalograms are then fed into a deep CNN encoder with adaptive squeeze-and-excitation (ASE), followed by a transformer and an adaptive gated feature fusion (AGFF) architecture. In this framework, to improve OSA detection performance, the CNN extracts rich local features, the ASE module performs channel-wise recalibration to enhance feature representations, the transformer performs data-parallel processing and captures global contextual dependencies, and the AGFF mechanism adaptively emphasizes informative features while suppressing less relevant ones. **Results:** The experimental results on the Apnea-ECG dataset showed that the model achieved a sensitivity of 94.7%, specificity of 95.2%, F1-score of 93.5%, accuracy of 95.1%, Cohen’s kappa of 89.4%, and an area under the receiver operating characteristic (ROC) curve (AUC) of 0.989 for per-segment classification. Furthermore, for per-recording classification, the model achieved an accuracy of 100.0%, a mean absolute error (MAE) of 2.025, and a Pearson correlation coefficient (PCC) of 0.992. Overall, the experimental results demonstrated that the proposed model achieved excellent and competitive performance compared with other advanced state-of-the-art methods for OSA classification. **Conclusions:** The proposed model demonstrates strong efficacy in OSA detection, providing a novel and robust alternative to conventional diagnostic methods. The model’s reliable and consistent diagnostic performance highlights its potential for integration into practical OSA diagnostic systems, including home-based health monitoring devices and clinical decision-support tools.

## 1. Introduction

Sleep apnea is categorized under sleep-related breathing disorders. It is characterized by repetitive interruptions in breathing during sleep, which can occur multiple times throughout a single night [1]. Obstructive sleep apnea (OSA), the more prevalent type, occurs due to the relaxation and collapse of the throat muscles, which partially (hypopnea) or completely (apnea) block airflow during sleep. Those apnea and hypopnea events lead to awakenings and fragmented sleep [1,2]. If OSA is left untreated, it can lead to various health-related symptoms, such as loud snoring, excessive daytime sleepiness, nonrestorative sleep, fatigue, and anxiety. In more severe cases, sleep apnea (SA) can be associated with serious cardiovascular and neurological sequelae, such as stroke, cardiocerebrovascular diseases, cardiopulmonary disorders, hypertension, arrhythmia, acute coronary syndrome, heart failure, and, in some cases, sudden death during sleep [1,3,4]. Benjafield et al. conducted a global analysis and found that OSA affects nearly one billion people worldwide, with prevalence rates of over 50% in some countries [5]. They found that 936 million adults aged 30–69 years have mild to severe OSA, while about 425 million adults aged 30–69 years have moderate to severe OSA globally [5]. These staggering statistics underscore the urgent need to implement efficient screening approaches to diagnose SA in a large population of potential patients within short period. Thus, the early identification of apnea and hypopnea events is of great importance, both in terms of patients health and the reduction in treatment costs.

In clinical practice, SA severity is assessed using the apnea-hypopnea index (AHI) or respiratory disturbance index (RDI) derived from polysomnography (PSG), the gold standard for SA diagnosis [6,7,8]. However, PSG is costly, subjective, and impractical for large-scale screening, leaving over 80% of high-risk individuals undiagnosed [7,9,10] and imposing an economic burden of $149.6 billion in productivity losses and comorbidity costs [11]. To address these limitations, in recent years, a growing number of studies have explored the use of physiological signals as alternative approaches for detecting SA, such as oxygen saturation SPO_2_ [12,13,14,15,16], airflow signals [17], EEG [18,19,20], and ECG [4,9,21,22,23,24,25,26,27,28,29,30,31,32,33,34]. Among these physiological signals, considerable attention has been directed toward ECG. ECG is the most widely used and accepted due to its rich information content, ease of acquisition, stable waveform morphology, and ability to capture cardiovascular responses linked to sleep-breathing events [24,35,36,37]. Thus, it is routinely and widely adopted in clinical practice for the evaluation and diagnosis of SA.

ECG-based approaches for OSA detection have developed into a significant and active area of research [4,9,21,22,23,24,25,26,27,28,29,30,31,32,33,34]. These approaches include direct analysis of raw ECG signals, feature-based methods that extract parameters such as R-peak amplitude (RA), RR intervals (RRI), ECG-derived respiration (EDR), statistical measures of heart rate variability (HRV), intrinsic mode functions (IMFs), as well as methods that transform ECG data into the time-frequency domain. The resulting data are then used as input to machine learning (ML) or deep learning (DL) models for SA event detection. Nevertheless, most existing approaches depend on extensive handcrafted feature extraction pipelines and treat temporal and spectral features independently, preventing the model from capturing the full multimodal cardiac dynamics associated with OSA episodes. CNN-based methods may have limited global dependency modeling, while existing approaches often lack feature recalibration and fusion, collectively compromising detection reliability across diverse patient populations. Motivated by these shortcomings, this study proposes SApneaNet to detect SA events from one-minute ECG segments and compute the AHI for estimating subject-level apnea severity over the entire recording for older adult patients. Inspired by recent advances in medical image analysis [38,39] and different from prior studies, this work introduces a novel hybrid CNN–Transformer architecture that leverages CWT-based RGB log scalograms to jointly encode the temporal and spectral ECG characteristics, enabling the model to focus on clinically relevant time-frequency features of ECG signals and potentially offering more interpretable representations for OSA detection. Thus, the SApneaNet offers a streamlined automated pipeline for robust and clinically reliable OSA detection.

## 2. Related Work

Recent traditional machine learning approaches for SA detection generally rely on manually generated features from ECG signals. From the resulting high-dimensional feature set, a subset of features is selected and classified using methods such as random forest (RF), linear discriminant analysis (LDA), support vector machine (SVM), K-nearest neighbors (KNN) and random under-sampling boosting (RUSBoost) [40,41,42,43,44,45]. For instance, Rajesh et al. proposed a discrete wavelet transform (DWT)-based OSA detection method, extracting statistical features from one-minute ECG segments, reduced via correlation-based feature selection (CFS)-particle swarm optimization (PSO) dimensionality reduction, and classified using LDA, KNN, SVM, and RF, with RF achieving the best accuracy of 90% [40]. Song et al. [41] proposed an OSA detection approach using RR intervals and EDR signals. Time and frequency domain features were extracted from these signals and classified using a discriminative hidden Markov model (HMM) combined with SVM. Their method achieved 97.1% accuracy for per-recording classification and 86.2% accuracy for per-segment detection [41]. In another study [43], Tripathy et al. proposed an approach for SA detection using the bivariate cardio-pulmonary (CP) signal. They decomposed the bivariate CP signal into IMFs using bivariate fast and adaptive empirical mode decomposition (FAEMD). They extracted 34 features from IMFs. These features were fed into SVM and RF classifiers to discriminate SA from normal events, achieving an average sensitivity and specificity of 82.27% and 78.67%, respectively [43]. Furthermore, Hassan et al. [44] proposed an expert system for identification of OSA. The authors decomposed one-minute ECG segments using the tunable-Q factor wavelet transform (TQWT), from which three statistical features were extracted. These features were then classified using random under-sampling boosting (RUSBoost) to differentiate non-apneic from apneic ECG segments. Their approach achieved 88.88% accuracy, 87.58% sensitivity, and 91.49% specificity on the Apnea-ECG dataset [44]. Although the abovementioned SA event detection methods show promising performance, they rely heavily on manually engineered features requiring substantial domain expertise, which are often dataset-specific and limit robustness and generalizability. The dependence on manual feature engineering also restricts scalability and automation, motivating the adoption of DL-based frameworks for fully automatic and knowledge-independent feature extraction.

In recent years, unsupervised DL methods have been investigated to extract informative representations from unlabeled data. For example, Li et al. [33] developed a methodology for SA detection that computed RR intervals from ECG signals and encoded them using a sparse autoencoder to automatically learn feature representations via an unsupervised learning approach that employs unlabeled ECG data. The extracted features were classified using SVM and ANN classifiers, while temporal dependencies were modeled via an HMM. A decision-level fusion strategy further improved performance. The method achieved 85% accuracy and 88.9% sensitivity for per-segment classification [33]. Similarly, in another study [32], the authors introduced an SA detection method in which RR intervals were extracted from ECG signals and fed into a frequential stacked sparse autoencoder to automatically learn features using an unsupervised approach. To address temporal dependence and class imbalance, a time-dependent cost-sensitive classifier was developed by integrating an HMM with the MetaCost algorithm. The method achieved per-segment classification performance of 85.1% accuracy, 86.2% sensitivity, and 84.4% specificity [32]. These approaches employed autoencoders for automatic feature extraction, reducing dependence on expert-designed features. However, their per-segment detection performance typically remained below 90% in terms of accuracy, sensitivity and precision, suggesting that unsupervised feature learning may not capture sufficiently discriminative information for effective SA event detection.

Given the ability of LSTM networks to learn temporal patterns from sequential data, studies have integrated CNN and LSTM architectures for SA event detection. For instance, Hossan et al. developed a model integrating multiple CNN–BiLSTM hybrid subnetworks in a cascaded structure for OSA detection. The results confirmed that decomposing the hybrid model into smaller subnetworks improves the representation of temporal dynamics [30]. Similarly, Kemidi et al. presented an OSA detection model named OSAD-Net. Deep features were first extracted from raw ECG signals using a multi-layer CNN [29]. An exhaustive feature selection strategy based on random forests was then employed to retain the most informative features. These selected features were subsequently used to train an optimized bidirectional LSTM for accurate OSA detection [29]. Moreover, Shao et al. performed OSA detection by extracting 1D linear and nonlinear HRV features alongside 2D time-frequency spectrum images from ECG signals. The 1D sequences were processed by a Bi-LSTM and the 2D images by a SqueezeNet CNN, with both outputs fused into a multilayer perceptron (MLP) for final OSA detection [31]. These studies employed LSTM or CNN + LSTM architectures and demonstrated the potential of DL for SA detection; however, they remain somewhat dependent on incomplete and subjective ECG feature sets, prone to gradient issues, and limited in parallel processing, resulting in high computational costs. This highlights the need for a model capable of capturing global dependencies while supporting parallelized processing.

Current studies have utilized CNN-based models for SA detection. For instance, Choudhury et al. transformed ECG signals into scalograms and fed them into a pre-trained GoogLeNet for transfer learning, employing LIME (Local Interpretable Model-Agnostic Explanations) for prediction interpretability [26]. Their method achieved 93.85% accuracy, with sensitivity, specificity, and F1-score of 93.42%, 94.30%, and 93.83%, respectively, on the Apnea-ECG dataset [26]. Zhou et al. investigated an OSA-Composite CNN for OSA detection using a hybrid image dataset comprising scalogram images with HRV features and gramian angular difference fields (GADF)-based 2D matrix images derived from ECG signals [27]. Three transfer learning sub-models (fine-tuned AlexNet, fine-tuned ResNet, and a custom CNN) were fused via a voting mechanism for final prediction, with the method designed for integration into wearable devices for continuous SA monitoring [27]. Mashrur et al. proposed a CNN for OSA detection by converting ECG signals into conventional and hybrid scalograms. For per-segment classification, the proposed method achieved 94.3% accuracy, while for per-recording classification, it achieved an accuracy of 100.0% [9]. Singh et al. investigated an approach for OSA detection using a pre-trained AlexNet model with transfer learning, where scalograms were used as input for classification [28]. Experimental results demonstrated that their proposed method achieved good performance while maintaining low computational cost and reduced complexity [28]. Collectively, the aforementioned studies using CNNs have demonstrated promising results by leveraging time-frequency images. However, CNNs are inherently limited to local feature extraction due to their receptive field, which restricts their ability to capture global features across an entire ECG segment. Although increasing the convolutional kernel size can expand the receptive field, excessively large kernels may introduce irrelevant information and reduce model performance. The majority of these approaches also lack an attention mechanism, causing all features to be treated equally; without attention, networks must be deeper, more complex, and heavier to highlight critical patterns.

Recent studies have employed attention mechanisms to focus on important features for sleep apnea (SA) event detection. For example, Hou et al. introduced the time-hybrid OSA transformer (THO), which extracted EDR, RRI, and RAMP features from ECG signals and enhanced them through temporal window-based LSTM augmentation. The fused features were then processed by CNN, LSTM, and transformer layers with time-based multihead attention (TBMA) to capture long-term dependencies, while a decoder and voting mechanism improved OSA prediction reliability [22]. Liu et al. investigated a hybrid CNN–Transformer model for OSA detection using raw single-lead ECG signals [25]. The CNN component extracted representative features, while the transformer captured long-range temporal dependencies. Their method achieved 88.2% accuracy and 0.95 ROC-AUC for per-segment classification, and 100% accuracy with a 4.33 mean absolute error (MAE) for per-recording classification, highlighting its potential for portable home-based OSA monitoring [25]. Furthermore, Hu et al. [4] proposed an OSA detection model in which raw ECG, RA, RRI, and RRID signals were processed using CNN blocks to extract feature representations. These features were subsequently processed by transformer blocks to capture the most relevant information via the self-attention mechanism. Their approach offered a practical solution for clinical OSA detection [4]. These CNN–Transformer studies using raw ECG signals have shown promising results. However, they primarily focus on time-domain information and often overlook the contribution of frequency-domain features to model performance. While raw signals and time-frequency images each have their own characteristics, time-frequency representations capture both time-domain and frequency-domain features. As a high-level representation of the raw signals, time-frequency images provide richer and more discriminative features, which can enhance CNN-based feature extraction and improve global dependency modeling with transformers. This motivates the integration of time-frequency images with CNN–Transformer architectures to improve SA event detection.

To overcome the aforementioned limitations, and different from prior studies, this study aims to develop a more accurate and robust approach for SA detection, addressing a key challenge in contemporary healthcare settings. Firstly, we use the CWT to transform ECG signals into RGB log-scalogram images, enabling the simultaneous extraction of time- and frequency-domain information. Secondly, we introduce SApneaNet, a novel adaptive squeeze-and-excitation-based CNN–Transformer network with adaptive gated feature fusion (AGFF), which integrates CNN, ASE, transformer, and AGFF modules within a unified framework. The uniqueness of SApneaNet lies in the incorporation of hierarchical CNN-based blocks, a newly designed ASE module, and a novel AGFF mechanism. In the SApneaNet model, each component serves a specific key role. The CNN layers first learn low-level feature representations, forming the basis for apnea event recognition. The ASE block performs channel-wise feature recalibration by emphasizing informative features and suppressing less relevant ones. Subsequently, the transformer block performs data-parallel processing, models global dependencies and mines high-level, rich feature representations that are essential for detecting intermittent apnea and hypopnea events characteristic of SA. Finally, the AGFF mechanism adaptively models the relative importance of CNN + ASE and transformer-derived features through a learnable gating strategy. This model was developed using five-fold cross-validation for hyperparameter selection on the training set and evaluated on an independent test set from the Apnea-ECG database, achieving excellent and competitive performance compared with SOTA methods. It effectively identifies subtle changes in ECG segments, simplifies the workflow, and demonstrates strong generalization capability across subjects, as well as robustness in physiological signal analysis, showing promising potential for effective SA detection. To the best of our knowledge, no previous study has proposed a unified framework integrating RGB log-scalograms with CNN, ASE, transformer, and AGFF modules for SA event detection. Overall, these developments represent a notable advancement in the field and open new opportunities for research and real-world applications.

Overall, the novelty and key contributions in this paper are as follows:

1. We propose a novel signal-processing pipeline that converts ECG signals into time-frequency RGB log scalogram images, providing a richer multi-channel representation. The ECG scalogram features are leveraged through advanced hierarchical CNN–Transformer framework, enabling effective learning of discriminative patterns for SA event detection.
2. We introduce a novel adaptive squeeze-and-excitation module for channel-wise feature recalibration and suppression of less relevant ones, along with a transformer block for modeling global feature dependencies via self-attention. In addition, a newly designed adaptive gated feature fusion (AGFF) mechanism is introduced to adaptively integrate CNN and transformer features for accurate prediction.
3. t-SNE-PSO and PCA provide interpretable visualizations of ECG segments, demonstrating clear separation between apnea and normal classes in the learned feature space, and revealing the structure of segment feature representations that underpin the model’s predictions. The method’s strong detection performance is confirmed through extensive experiments on the Apnea-ECG dataset.

The rest of this paper is organized as follows. Section 3 describes the datasets, processing steps, and proposed method. Section 4 provides the evaluation metrics, hyperparameter settings, experimental results, and ablation studies. Section 5 reports comparative performance experiments, analyzes robustness and generalizability, and discusses future directions. Finally, Section 6 presents the conclusions of this article.

## 3. Materials and Methods

### 3.1. Data Description

In this study, the proposed method was trained and evaluated using the Apnea-ECG Database [46], which is publicly available from PhysioNet [47] at https://www.physionet.org/content/apnea-ecg/1.0.0/ (accessed on 8 October 2025). The Apnea-ECG database consists of 70 overnight single-lead ECG recordings acquired using a modified lead V2 electrode configuration, with a sampling frequency of 100 Hz and 16-bit resolution. Among the 70 subjects, 57 were male and 13 were female, aged 27 to 63 years (45.1 ± 10.8 years). Their weights ranged from 53 to 153 kg (86.8 ± 20.6 kg), with heights from 158 to 184 cm (175.8 ± 5.5 cm), corresponding to body mass indices (BMI) between 19.2 and 45.3 (28.0 ± 6.4 kg/m^2^). Those overnight single-lead ECG recordings have durations ranging from 401 to 578 min (492 ± 31 min), corresponding to approximately seven to ten hours. Each ECG recording in the dataset is manually annotated at one-minute intervals by sleep medicine specialists. The dataset is impartially divided into 35 subjects for the training set (indices a01–a20, b01–b05, and c01–c10) and 35 subjects for the test set (indices x01–x35). The training set contains 17,125 one-minute ECG segments (6514 apnea and 10,611 normal), while the test set contains 17,303 one-minute segments (6552 apnea and 10,751 normal). Subjects have AHI values that range from 0 to 93.5. Based on the AHI criterion, the dataset includes 46 apneic subjects and 24 normal subjects; where the training set contains 23 recordings from SA subjects and 12 recordings from normal subjects, while the test set contains 23 recordings from SA subjects and 12 recordings from normal subjects. Table 1 summarizes the PhysioNet Apnea-ECG dataset used in this study. The dataset exhibits class imbalance, consisting of 34,428 one-minute ECG segments, of which 13,066 (38%) are apneic and 21,362 (62%) are normal, indicating a substantial underrepresentation of apnea segments.

### 3.2. Data Preprocessing

**Figure 1:**
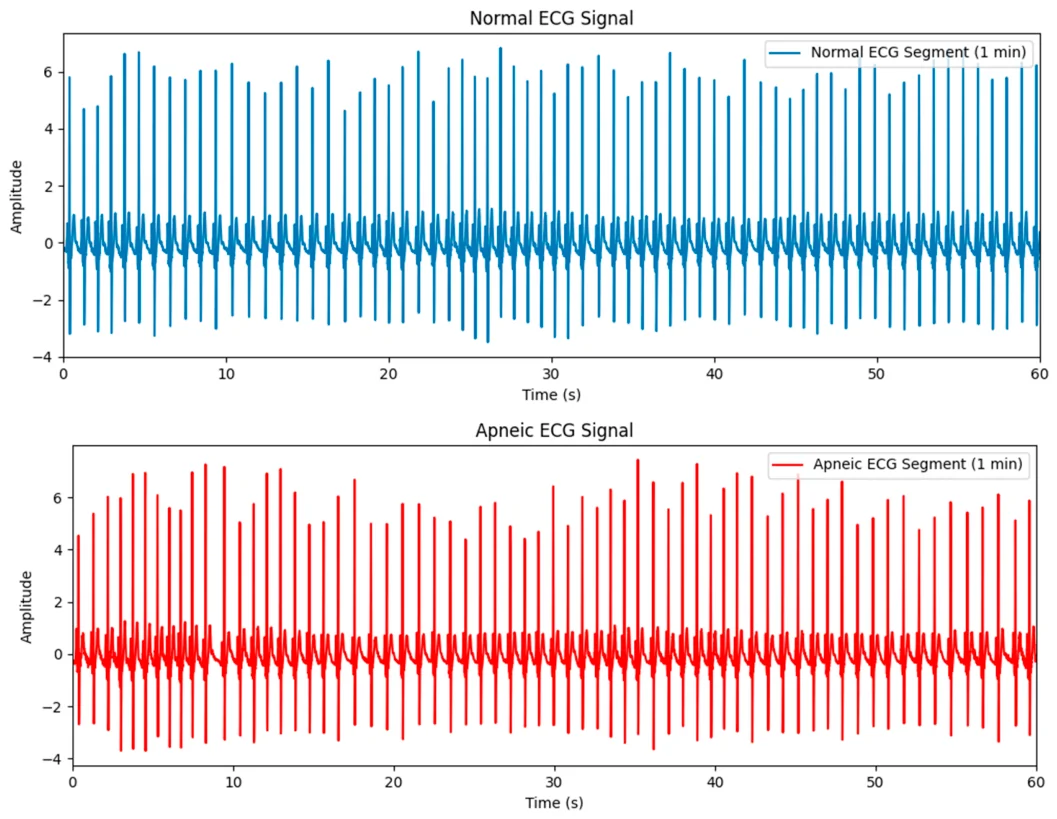
Representative one-minute normal and apnoeic ECG segments. Differences in R–R interval variability and heart rate variability (HRV) between the two segment types can be subtle in the raw time-domain waveform and are not always readily discernible by visual inspection alone. This motivates the transformation of ECG signals into the time-frequency domain (Section 3.4 and Figure 2), where such variations can become more distinguishable, as illustrated by the corresponding RGB log-scalogram representations in Figure 3.

ECG signals are inherently nonstationary and stochastic due to the complex electrophysiological dynamics of the human body. The original ECG signal acquired by ECG devices is frequently corrupted by various noise sources and artifacts, resulting in signal quality degradation and limiting reliable interpretation and feature extraction, particularly for SA event detection. These signals typically contain both low-frequency noise, such as baseline wander, and high-frequency noise, including electromyographic (EMG) and power-line interference. Therefore, in this study, robust filtering and denoising techniques are essential to ensure accurate ECG analysis. We employed a second-order Butterworth bandpass filter with cutoff frequencies of 0.5 Hz and 40 Hz [25] to remove low- and high-frequency noise components in the ECG signal. This approach eliminates baseline drift and power-line interference while preserving the essential morphology of the original ECG signal. The denoised ECG signals are standardized to reduce inter-patient variability. However, long-segment ECG recordings may contain occasional false spikes caused by artifacts, which can distort min–max normalization and make it unreliable. To address this issue, Z-score normalization is employed, as it provides a more robust normalization approach by reducing the influence of such outliers, as defined in Equation (1).

z(t) = (s(t) − μ)/σ(1)

where $s\left(t\right)$ denotes the original ECG signal, μ and σ are the mean and standard deviation of $s\left(t\right)$, respectively, and z(t) represents the normalized signal. After denoising and standardization, the ECG signals were segmented into one-minute intervals to align with the per-minute annotations provided by sleep medicine specialists, where each segment consists of 6000 samples. Since the overnight ECG recordings are segmented into one-minute intervals, the segmentation module verified that each one-minute segment contained exactly 6000 samples; therefore, any residual segment containing fewer than 6000 samples was automatically discarded by the segmentation module. Consequently, 157 incomplete segments were removed from the original 34,428 segments, resulting in 34,271 complete one-minute ECG segments. This segmentation facilitates subsequent data processing and accurate label correspondence, thereby improving SA detection performance. After preprocessing, a total of 34,271 segments were obtained, with 17,023 segments (6511 SA and 10,512 normal) assigned to the training set and 17,248 segments (6547 SA and 10,701 normal) to the test set. Figure 1 shows representative preprocessed ECG segments from both classes.

**Figure 2:**
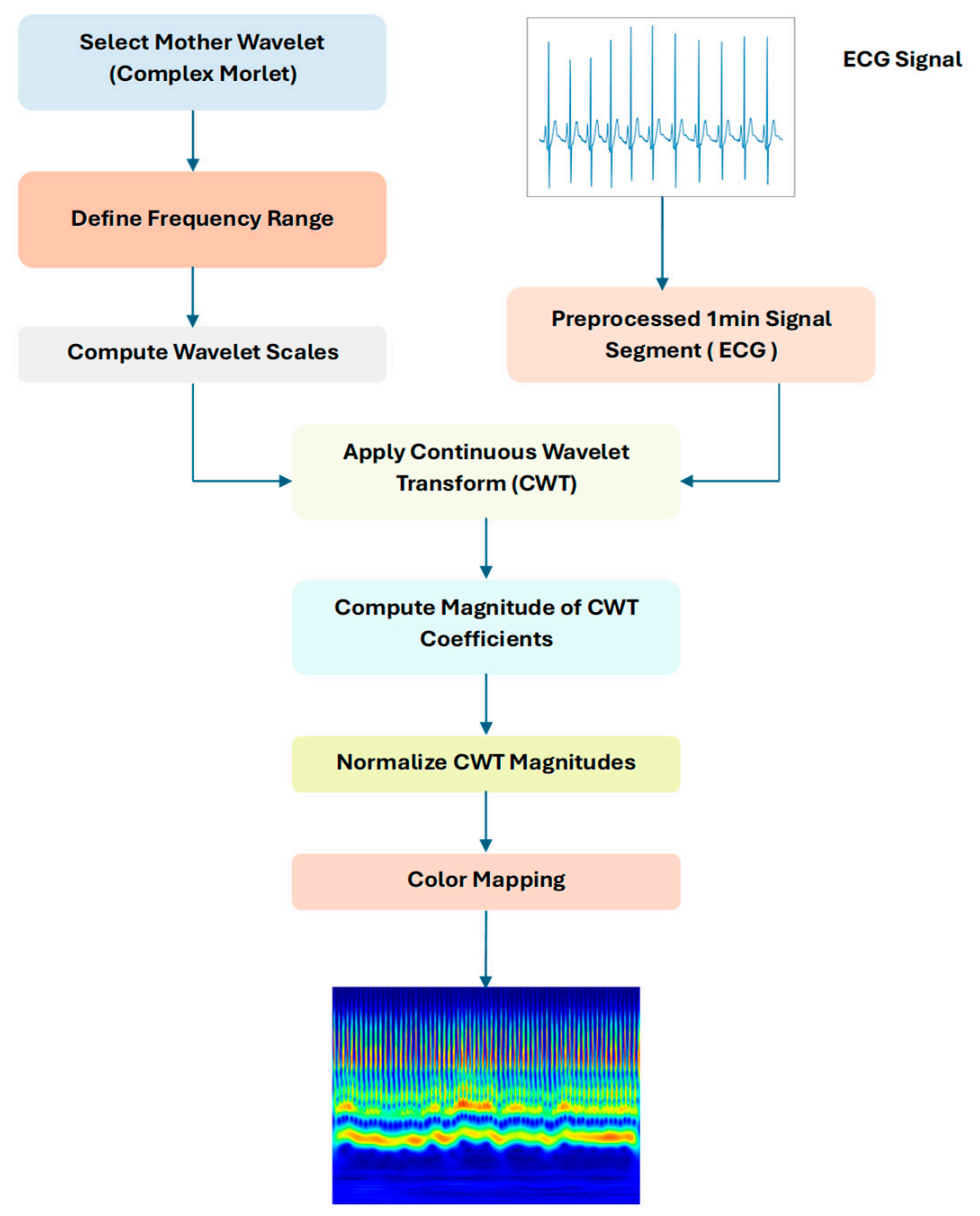
Proposed CWT-based RGB log-scalogram generation pipeline for ECG signal representation.

**Figure 3:**
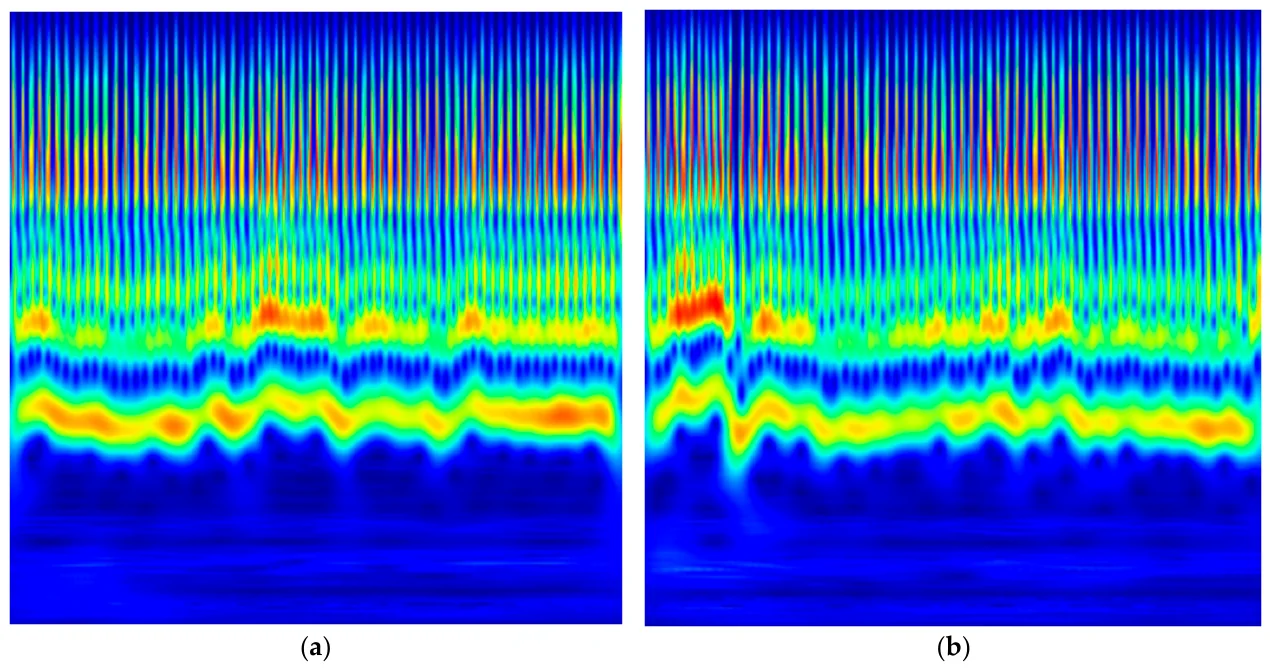
RGB log-Scalograms of normal and apneic segments. (**a**) Normal ECG Scalogram. (**b**) Apneic ECG Scalogram.

### 3.3. Signal Transformation into Time–Frequency Image Using the Continuous Wavelet Transform (CWT)

ECG signals are integrally non-stationary, and representing them solely in the time or frequency domain does not capture all their information [48]. Therefore, this study performs a detailed time-frequency analysis of ECG signals using the CWT to generate time-frequency images called RGB log scalograms, which capture the time-frequency characteristics of the signal. The CWT of a signal ϕ(t) with respect to a wavelet ψ(t) is obtained by computing the inner product between the signal and scaled and translated versions of the wavelet. Mathematically, it is defined in Equation (2):

(2) $$X_{\phi }^{\psi }\left(s,\tau \right)=\left\langle \phi ,\psi_{s,\tau }\right\rangle =\frac{1}{\sqrt{s}}\int_{-\infty }^{+\infty }{\phi \left(t\right)\;}^{.}\;\psi^{*}\left(\frac{t-\tau }{s}\right)dt$$

where ϕ(t) represents an ECG signal segment, and $\psi_{s,\tau }(t)$ is the scaled and translated version of the mother wavelet. The translation parameter τ shifts the wavelet along the time axis t, and the scale parameter s compresses or dilates the wavelet to adjust time-frequency resolution. The resulting wavelet coefficients $X_{\phi }^{\psi }(s,\tau )$ describe the signal segment ϕ(t). The magnitude scalogram is obtained as the absolute value of these coefficients $|X_{\phi }^{\psi }(s,\tau )|$, which represents the amplitude distribution of the signal over time and frequency. Compared to discrete wavelet transform (DWT), short-time Fourier transform (STFT), Wigner-Ville transform (WVT), and Choi-Williams distribution (CWD), the CWT is effective for capturing discriminative features of SA events due to its ability to represent non-stationary ECG signals with adaptive multi-resolution time-frequency analysis [49]. It provides clean scalogram representations by avoiding cross-term interference and preserves temporal resolution across frequency bands. This makes it well-suited for characterizing rapid transient dynamics in ECG signals associated with apnea events, enabling more accurate SA event detection [49].

### 3.4. RGB Log-Scalogram Generation

The construction of log scalograms as time-frequency representations constitutes a key component of time-frequency analysis. In this study, scalograms are generated using the CWT. The CWT is applied to transform each one-minute ECG segment (ϕ) into an ECG log scalogram. The analysis begins with the selection of a suitable mother wavelet; in this work, the complex Morlet wavelet is used due to its strong similarity to ECG signals, effectively represents complex-valued spectro-temporal features, and generates high-resolution scalogram representations [50]. In this study, the complex Morlet wavelet family is generated from the following mother wavelet in Equation (3):

(3) $$\left(t\right)=\frac{1}{\sqrt{\sigma_{t}\sqrt{\pi }}}e^{-t^{2}/\left(2\sigma_{t}^{2}\right)}e^{j2\pi f_{c}t}$$

In this formulation, $e^{j2\pi f_{c}t}$ represents a complex sinusoidal oscillation at the center frequency $f_{c}$, while $e^{-t^{2}/(2\sigma_{t}^{2})}$ is a Gaussian window (envelope) with temporal standard deviation $\sigma_{t}$. The normalization factor $1/\sqrt{\sigma_{t}\sqrt{\pi }}$ ensures that the wavelet has unit energy.

Then, the target frequencies are first specified to determine the scales for wavelet analysis. Using a complex Morlet wavelet, the target frequencies were sampled on a logarithmic scale with 48 voices per octave. These frequencies were converted into logarithmically spaced scales using the wavelet’s central frequency and the signal’s sampling rate. CWT coefficients are obtained by convolving the scaled and shifted wavelets with the segment, producing a structured representation that links signal dynamics to the corresponding time and scale points. Finally, the CWT coefficients are converted to their absolute values, logarithmically transformed using $S_{log}\left(t,f\right)=10{log}_{10}\left(|W\left(t,f\right)|+\varepsilon \right)$, where $\varepsilon =10^{-10}$ is a small constant added for numerical stability, and normalized to the range [0,1]. These values are then mapped to a 256-color colormap to generate a scalogram image of size 64 × 64 × 3, in which variations in magnitude across time and scale are visually color-encoded, highlighting abrupt changes and subtle patterns that may not be evident in the original signal. Notably, differences between normal and apnea-affected segments become apparent, as shown in Figure 3: normal signals exhibit smoothly varying color patterns, whereas signals during apnea show sudden, sharp variations. Such image representations effectively convert temporal information into a rich visual format, facilitating the identification and distinction of pathological events from normal activity. Figure 2 illustrates the proposed CWT-based RGB log-scalogram generation pipeline, while Figure 3 presents representative RGB log-scalograms of normal and apnea-affected ECG segments.

### 3.5. Proposed SApneaNet Architecture: CNN-ASE–Transformer with AGFF

**Figure 4:**
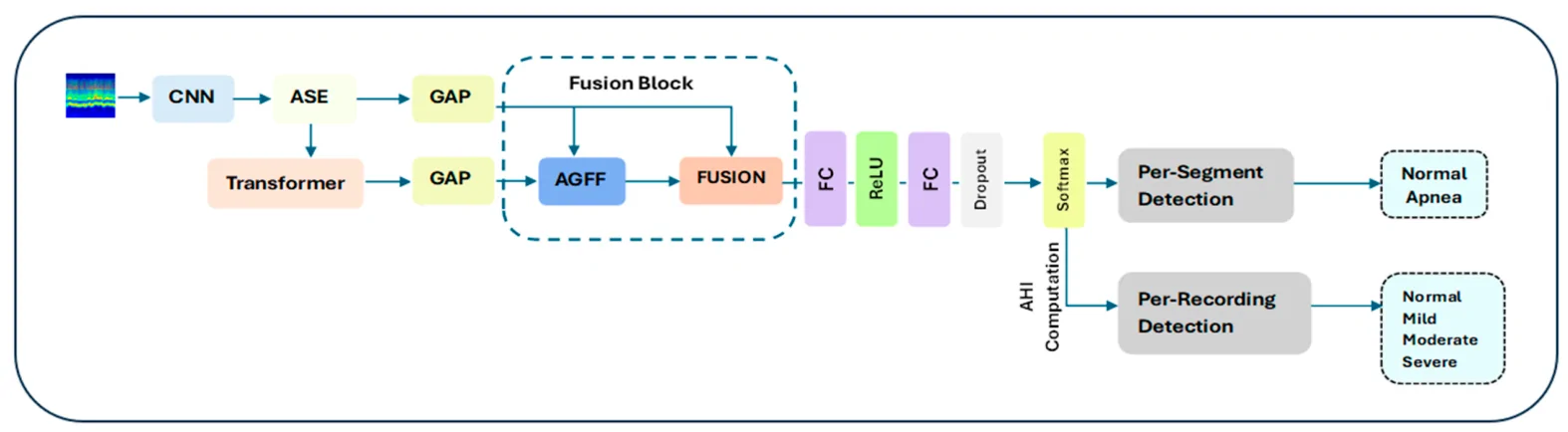
Architecture of the proposed SApneaNet model for SA detection using ECG images.

In this study, we propose SApneaNet, a novel advanced CNN-ASE-Transformer-with-AGFF-based framework for SA event detection. The model leverages RGB log-scalogram representations derived from ECG signals and enables complementary feature learning through CNN-ASE and transformer (AGFF) branches, facilitating the effective extraction of discriminative time-frequency features beyond conventional bio-signal image-based approaches. The proposed SApneaNet architecture is designed for both per-segment apnea event detection and per-record SA severity screening (normal, mild, moderate, and severe). Unlike prior studies, the proposed framework exploits complementary representations derived from RGB log-scalogram images through CNN-ASE-Transformer architecture integrated with an AGFF mechanism, as illustrated in Figure 4. The model processes RGB log-scalogram images to capture time-frequency morphological features and oscillatory cardiac patterns. The CNN-ASE branch is responsible for local feature extraction by learning spatial representations enriched with channel-wise adaptive recalibration, whereas the transformer branch models global contextual relationships by learning dependencies among feature tokens to optimize feature representations. The CNN-ASE and transformer-derived features are separately aggregated using global average pooling (GAP). The AGFF module then adaptively emphasizes informative features while suppressing less relevant ones. The resulting high-level features are subsequently fused and fed into a decoder block comprising dense layers with ReLU activation, a dropout layer for regularization, and a final dense layer with softmax activation function for accurate apnea event detection at both segment and record levels. This multi-stage hybrid representation learning framework enables the SApneaNet to leverage the complementary time-frequency morphological characteristics encoded in RGB log-scalograms, thereby enhancing robustness and classification performance.

(1). Convolutional Neural Networks (CNNs)

**Figure 5:**
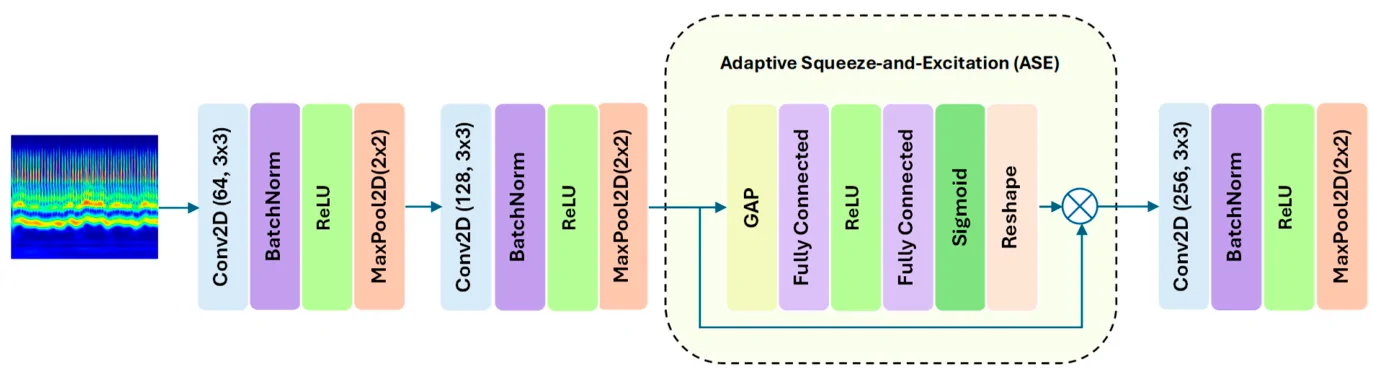
Architecture of CNN with Adaptive Squeeze-and-Excitation (ASE) module.

In this work, CNNs are employed to extract spatially organized spectral features from ECG-derived images while progressively transforming and compressing the input across feature-map channels, which are then fed to subsequent network components, such as ASE and transformers for further processing. The model consists of convolutional blocks; through successive convolution and pooling operations, the model learns hierarchical representations from the input image while preserving salient information relevant for SA detection. The architecture of the CNN and its parameters are illustrated in Figure 5 and Table 2, respectively.

(2). Adaptive Squeeze-and-Excitation (ASE) Module

To enhance discriminative feature learning from the RGB log scalogram representations, an adaptive squeeze-and-excitation (ASE) module is incorporated within the CNN architecture. The ASE module adaptively recalibrates channel-wise feature responses by modeling inter-channel dependencies, enabling the network to emphasize informative feature channels while suppressing less relevant ones. Specifically, the module first applies global average pooling (GAP) to generate compact global channel descriptors through a squeeze operation. These descriptors are subsequently passed through two parallel fully connected pathways with nonlinear activation to learn adaptive channel-wise importance weights, while a separate mixture-weight sub-network dynamically generates sample-dependent weights for combining the two excitation pathways. The resulting excitation weights are then applied to the original feature maps through channel-wise multiplication, allowing the network to selectively strengthen salient ECG-related time-frequency patterns associated with SA events. By dynamically enhancing relevant feature representations, the ASE module improves the discriminative capability and expressivity of the CNN-extracted time-frequency features, facilitating more robust feature learning and improved SA event recognition performance. The ASE computation is formally expressed in Equation (4) and is illustrated in Figure 5.

(4) $$\hat{X}=X⊗\;\left[\sum_{k=1}^{2}\frac{\exp\left({\left(W_{\alpha }z+b_{\alpha }\right)}_{k}\right)}{\sum_{j=1}^{2}\exp\left({\left(W_{\alpha }z+b_{\alpha }\right)}_{j}\right)}\cdot \sigma \left(W_{2}^{\left(k\right)}\;\delta \left(W_{1}^{\left(k\right)}z+b_{1}^{\left(k\right)}\right)+b_{2}^{\left(k\right)}\right)\;\right]\;,\;z=GAP(X)$$

where δ denotes the ReLU activation, σ denotes the sigmoid function, ⊗ denotes channel-wise multiplication, and $r_{1}=4$, $r_{2}=8$ are the fixed reduction ratios of the two excitation pathways. $W_{1}^{\left(k\right)},W_{2}^{\left(k\right)},b_{1}^{\left(k\right)},b_{2}^{\left(k\right)}$ are the learnable weights and biases of pathway k, and $W_{\alpha },b_{\alpha }$ are the learnable parameters of the mixture-weight sub-network that produces the sample-dependent mixture weights.

(3). Transformer

**Figure 6:**
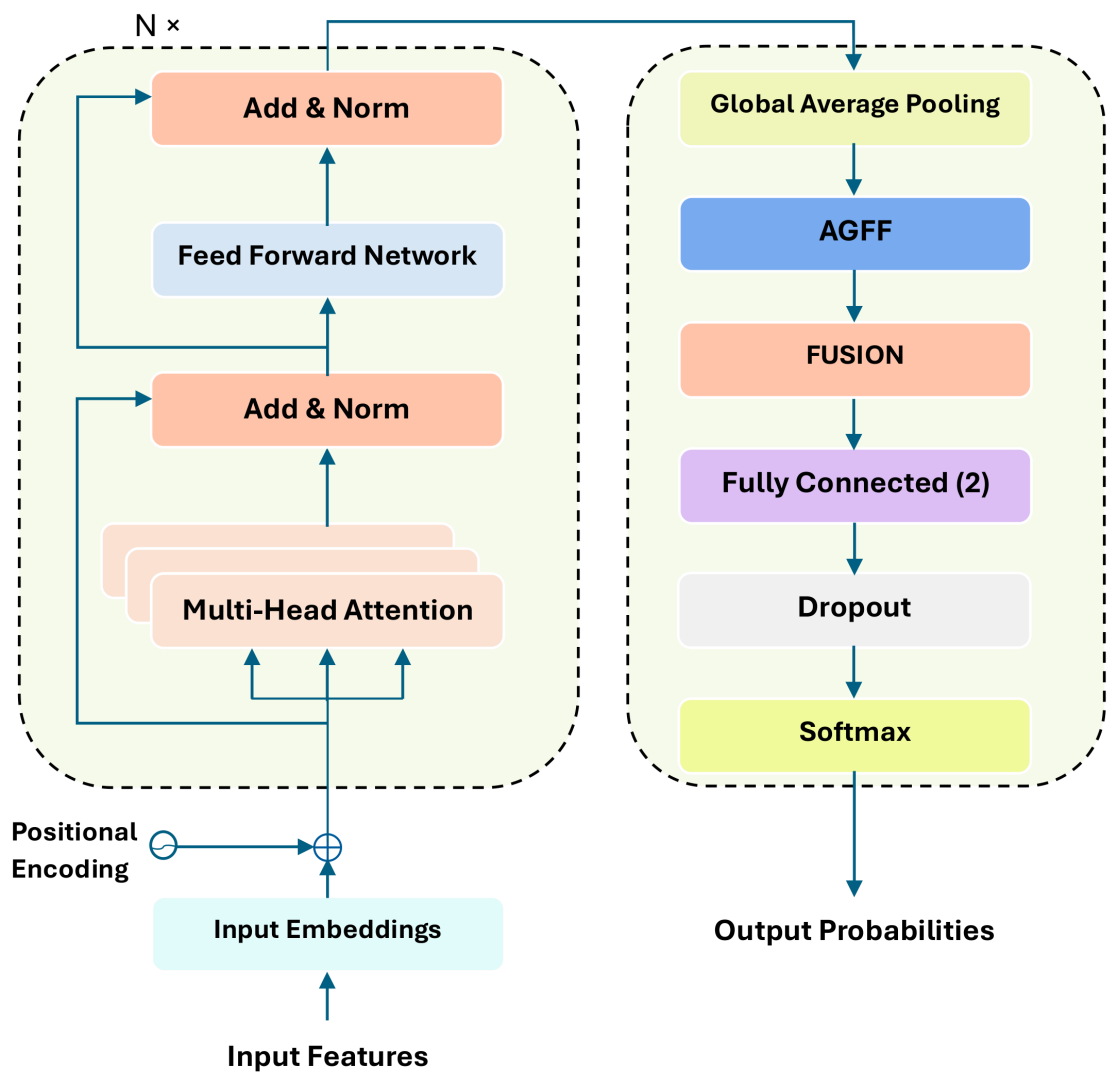
Transformer encoder architecture with AGFF-based fusion and classification head.

The third component of the SApneaNet consists of a transformer with a self-attention mechanism [51,52], which is used to model relationships among feature tokens. In ECG-based analysis, intra-segment representations derived from RGB log-scalogram features exhibit meaningful contextual dependencies. Therefore, in this study, a transformer is employed within SApneaNet to model relationships among time-frequency features extracted by the CNN-ASE block. Unlike traditional RNNs and LSTMs, the transformer captures intra-segment dependencies through self-attention rather than sequential processing, enabling more efficient contextual modeling [53]. For each input segment, tokens are obtained via a convolutional token-embedding operation [54,55], in which an overlapping strided convolution (kernel 3 × 3, stride 2, 128 filters) is applied to the CNN-ASE feature map. The resulting feature map is reshaped into a token feature matrix $O\in R^{T\times F}$ with T=16 tokens of dimension F=128 each. Fixed sinusoidal positional encodings [51] are added to preserve token order, resulting in the final representation $\tilde{O}=O+PE$ [51,52], which serves as the input to the transformer encoder. Unlike ViT’s non-overlapping patch projection [56], SApneaNet employs convolutional tokenization within a hybrid CNN–Transformer architecture with a flat transformer processing strategy [57]. As shown in Figure 6, the transformer encoder refines these representations by capturing intra-segment dependencies among feature tokens, enabling more effective representation learning. The transformer encoder consists of two core modules: a multi-head self-attention (MHSA) module and a position-wise feed-forward network (see Figure 6). The MHSA computes attention using query (Q), key (K), and value (V) projections, as expressed in Equations (5) and (6), enabling the model to learn contextual dependencies among feature tokens [51,52].

(5) $$X_{i}=Attention\left(Q_{i},K_{i},V_{i}\right)=Softmax\left(\frac{Q_{i}K_{i}^{T}}{\sqrt{F/H}}\right)V_{i}$$

(6) $$M_{s}=Concat\left(X_{1}{,X}_{2},X_{3},...,X_{H}\right)W^{s}$$

The encoder also includes residual connections and normalization layers as illustrated in Figure 6, which help stabilize training and accelerate convergence. The second key component of the encoder is an FFN, which applies nonlinear transformations to the MHSA output and extracts salient features. The FFN consists of two fully connected layers with a ReLU activation function in between, as expressed in Equation (7):

(7) $$Z_{FFNN}=ReLU\left(L_{N}W_{1}+b_{1}\right)W_{2}+b_{2}$$

In this study, two transformer encoder layers were employed, each consisting of MHSA and FFN. The number of attention heads influences model performance, where H=1 limits the ability to capture diverse feature interactions, while H=4 or H=8 excessively fragments the feature space, reducing effective information integration and degrading performance. Based on empirical evaluation, H=2 provided a balanced representation capacity, enabling more effective feature interactions and yielding the highest accuracy. The FFN contains 256 hidden units, and a dropout rate of 0.2 was applied to both the self-attention and feed-forward layers. Overall, the transformer encoder receives feature representations extracted by the CNN-ASE block and models dependencies among feature tokens within each segment, producing enhanced representations for subsequent processing.

(4). Adaptive Gated Feature Fusion (AGFF) Mechanism

Finally, after the feature extraction and optimization stages, high-level feature representations are obtained through CNN-ASE and transformer encoding stages. Specifically, the CNN-ASE and transformer-derived features are separately processed using GAP as shown in Figure 4, yielding GAP-pooled feature vectors $f_{c}\in R^{d_{c}}=GAP(F_{CNN-ASE})$ and $f_{t}\in R^{d_{t}}=GAP(F_{Transformer})$. This GAP operation is used as a replacement of the traditional flatten–fully connected approach, as it reduces spatial dimensionality while preserving global context and improving robustness to spatial variations without introducing additional trainable parameters. To effectively integrate these heterogeneous feature representations, this work introduces an adaptive gated feature fusion (AGFF) strategy, unlike prior studies that rely on conventional concatenation-based fusion. The AGFF employs a learnable gating mechanism that adaptively models the relative importance of CNN-ASE and transformer features. Specifically, a sigmoid-activated dense layer generates feature-wise gates from the concatenated representations, which are then used to recalibrate the transformer feature branch through element-wise multiplication. This adaptive modulation allows the network to dynamically emphasize more informative features, particularly discriminative apnea-related patterns, while suppressing less relevant ones. The refined (recalibrated) transformer features are then concatenated with CNN-ASE features to form a unified embedding and subsequently passed through a stack of fully connected layers with ReLU activation for nonlinear feature integration. A dropout rate of 0.4 is applied to reduce overfitting, and finally a softmax classifier is used to categorize sleep breathing events into normal and apnea classes. The AGFF computation is formally expressed in Equation (8), which explicitly captures the input-dependent gating, transformer feature recalibration, and the subsequent feature fusion.

(8) $$f_{FUSION}=\left[f_{c};\sigma (W_{2}\delta (W_{1}\left[f_{c};f_{t}\right]+b_{1})+b_{2})⊙f_{t}\right]$$

where σ denotes the sigmoid function, δ denotes the ReLU activation, ⊙ denotes element-wise multiplication, $\left[\cdot ;\cdot \right]$ denotes vector concatenation, and $W_{1}$, $W_{2}$, $b_{1}$, $b_{2}$ are the learnable weights and biases of the two-layer gating MLP.

## 4. Experiments and Results

### 4.1. Parameters Setting

Experimental performance is highly related to the model hyperparameters. Therefore, the proposed SApneaNet was optimized by carefully tuning its key hyperparameters to achieve the best possible performance. The final model was then trained using the selected optimal hyperparameter configuration. In this study, the dataset was first divided according to the official Apnea-ECG database split into a training set comprising 35 subjects (a01–a20, b01–b05, and c01–c10) and an independent test set comprising 35 subjects (x01–x35), with no overlap of subjects between the two sets. To select the optimal hyperparameters, a 5-fold cross-validation strategy was employed exclusively within the 35 training subjects to ensure an objective and comprehensive assessment. In each iteration, one fold was used as the validation set while the remaining folds were used for training, resulting in five training and validation runs, where each fold served as the validation set once. All segments from the same subject were kept within the same fold to enhance the robustness and generalization of the model while preventing subject-level data leakage between the training and validation folds. During cross-validation, the model was evaluated on the validation fold in each iteration, and the validation predictions from the five folds were aggregated to assess model performance. The independent 35-subject test set remained completely untouched during training, validation, and hyperparameter tuning. The performance results reported in Table 3, Table 4, Table 5 and Table 6 were obtained exclusively from the independent 35-subject test set. The model was optimized using the Adam optimizer, an extension of stochastic gradient descent (SGD), which was chosen for its efficiency in handling sparse gradients and its adaptability to large datasets. The learning rate was set to *α* = 0.0001 to control the convergence speed and reduce the risk of convergence to local minima. The exponential decay rates for the first and second moment estimates were set to *β*_1_ = 0.9 and *β*_2_ = 0.999, respectively, along with a small constant *ε* = 1 × 10^−8^ for numerical stability. The categorical cross-entropy was used as the loss function, and L2 regularization with a weight decay of λ = 0.0001 was applied to reduce overfitting. The model used 25 training epochs with data shuffling before each epoch, providing a balanced trade-off between learning and performance. The ReduceLROnPlateau function was used to adaptively reduce the learning rate when performance stagnated, while the EarlyStopping function was applied to prevent overfitting by terminating training if the validation loss failed to improve for 10 consecutive epochs. The batch size was set to 64 to balance global information processing and hardware limitations.

### 4.2. Performance Evaluation Metrics

In this study, we evaluated both per-segment SA detection and per-recording SA detection, referring to the performance evaluation methods of other recent studies [4,9,21,22,23,25,30,31,32,33,40,41,43,44,50].

(1). Per-Segment SA Detection

This approach involves dividing long-term ECG recordings into one-minute segments. Each segment is then assigned the annotation provided by sleep experts, labeled as “normal (0)” or “apnea (1)”. The SApneaNet is trained on these labeled segments and subsequently used to predict the classification of one-minute segments in new, unseen subjects. The performance of our model is evaluated using widely adopted metrics from recent studies, allowing for a thorough evaluation of its strengths and limitations while enabling a fair comparison with prior work. We calculated true positives (TPs), false positives (FPs), true negatives (TNs), and false negatives (FNs), which were then used to derive the confusion matrix (CM), sensitivity (Sen), specificity (Spe), positive predictive rate (Ppr), Fβ−score, accuracy (Acc), Cohen’s kappa (κ) [4,22,26,32] and Matthews correlation coefficient (MCC) [4,24,26,30]. The mathematical formulations of these metrics are presented below.

(9) $$Ppr=\frac{TP}{TP+FP}$$

(10) $$Sen=\frac{TP}{TP+FN}$$

(11) $$Spe=\frac{TN}{TN+FP}$$

(12) $$F\beta -score=(1+\beta^{2})\times \frac{Ppr\;\times \;Sen}{(\beta^{2}\;\times \;Ppr)+Sen}$$

(13) $$Acc=\frac{TP+TN}{TP+TN+FP+FN}$$

(14) $$P_{o}=Acc=\frac{TP+TN}{TP+TN+FP+FN}$$

(15) $$P_{e}=\frac{\left(TP+FP)(TP+FN)+(TN+FP)(TN+FN\right)}{{\left(TP+TN+FP+FN\right)}^{2}}$$

(16) $$\kappa =\frac{P_{o}-P_{e}}{1-P_{e}}$$

(17) $$MCC=\frac{TP\times TN-FP\times FN}{\sqrt{\left(TP+FP)(TP+FN)(TN+FP)(TN+FN\right)}}$$

In this study, β is assigned a value of 1 to equally weight recall and precision. The Apnea class is treated as the positive class (1), and the normal class as the negative class (0). TP represents the number of apneic segments correctly predicted as apnea, TN denotes the number of normal segments correctly classified, FP is the number of normal segments incorrectly predicted as apnea, and FN represents the number of apneic segments incorrectly classified as normal.

The receiver operating characteristic (ROC) curve is also employed to evaluate the performance of the model across different classification thresholds, illustrating the trade-off between the true positive rate (TPR) and the false positive rate (FPR) [58], where a curve closer to the upper-left corner indicates better classification performance. Furthermore, the area under the ROC curve (AUC) is computed to quantify the model’s overall discriminative capability, with higher values indicating better separability between the apnea and non-apnea classes and thus superior detection performance [58]. Given the class imbalance in the segment-level data, the precision–recall (PR) curve is additionally employed to evaluate the trade-off between precision and recall across classification thresholds, with a curve closer to the upper-right corner indicating better performance [59]. The corresponding area under the PR curve (PR-AUC) is computed to provide complementary information to ROC-AUC by focusing on the positive (apneic) class under class-imbalanced conditions [59]. To complement these AUC-based global measures, we also introduced Youden’s index (J) to capture the optimal trade-off between correctly detecting apnea events and minimizing false alarms, which is useful for selecting the optimal threshold for practical deployment of the model. Youden’s index combines sensitivity (correct detection of apnea events) and specificity (correct identification of non-apnea events while minimizing false alarms) [60]. The index ranges from 0 to 1, where higher values indicate better diagnostic performance and a better balance between detecting apnea events and rejecting non-apnea events. Youden’s index is computed as expressed in Equation (18) [60]:

(18) $$J=\left(\frac{TP}{TP+FN}\;+\frac{TN}{TN+FP}\right)-1=\left(Sensitivity+Specificity\right)-1$$

(2). Per-Recording SA Detection

For individual-level SA screening, we evaluated SA using the apnea-hypopnea index (AHI), defined as the ratio of the total number of apnea and hypopnea events to the total hours of sleep. AHI is the clinical gold-standard metric defined by the guidelines of the AASM, Darien, IL, USA (1999); where clinically, a person is diagnosed with SA when the AHI exceeds 5 events per hour; otherwise, the subject is considered normal (healthy subject) [6]. In this study, the occurrence of SA events is approximated from minute-segmented ECG data, where each ECG segment classified as SA by the SApneaNet is counted as one apnea-hypopnea minute. For each subject, the number of apneic segments in the ECG recording is counted, and an estimated apnea-hypopnea index (AHI) is calculated to approximate the expected number of apnea and hypopnea events per hour. If AHI ≥ 5, the subject is classified as SA-positive; otherwise, the subject is considered SA-negative. This approach allows the conversion of per-segment classification results into a single per-recording diagnosis, enabling both screening and grading of SA at the individual level using single-lead ECG recordings for healthy or apneic subjects. The mathematical formula for AHI is defined in Equation (19).

(19) $$AHI=\frac{60}{T_{s}}\times \sum_{Record}N\left({Apnea}_{1-min}\right)$$

where $\sum_{Record}N\left({Apnea}_{1-min}\right)$ represents the total number of one minute with apnea events in the particular ECG recording, and $T_{s}$ denotes the total duration of the recording in minutes. This minute-based formulation approximates the clinical AHI, since the ground-truth annotations of the Apnea-ECG database are provided at one-minute resolution; this approximation is consistent with the approach used in the prior studies evaluated on this dataset. The estimated AHI reflects the average number of minutes per hour that a subject experiences SA. When a subject is found to have SA events, the severity of the condition is assessed using the AHI value. According to the clinical classification standard, an AHI<5 is considered normal, 5≤AHI<15 indicates mild SA, 15≤AHI<30 indicates moderate SA, and AHI≥30 corresponds to severe SA [6]. The individual-level diagnostic outcomes produced by the SApneaNet, which classify patients’ condition severity, are presented under the "OSA Severity Assessment" list item.

Furthermore, to evaluate per-recording SA detection, the predicted AHI values were compared with the corresponding ground-truth AHI values. To this end, we introduced two evaluation metrics, namely the mean absolute error (MAE) and the Pearson correlation coefficient (PCC). The MAE was used to assess the magnitude of prediction errors, while the PCC was calculated to evaluate the linear relationship between the predicted and true AHI values. Reporting both metrics provides a comprehensive view of the SApneaNet’s performance. The MAE and PCC are mathematically defined in Equations (20) and (21), respectively.

(20) $$MAE=\frac{1}{M}\sum_{j=1}^{M}\left|{AHI}_{j}^{pre}-{AHI}_{j}^{true}\right|$$

(21) $$PCC=\frac{\sum_{j=1}^{M}\left({AHI}_{j}^{true}-{\bar{AHI}}^{true}\right)\left({AHI}_{j}^{pre}-{\bar{AHI}}^{pre}\right)}{\sqrt{\sum_{j=1}^{M}{\left({AHI}_{j}^{true}-{\bar{AHI}}^{true}\right)}^{2}}\sqrt{\sum_{j=1}^{M}{\left({AHI}_{j}^{pre}-{\bar{AHI}}^{pre}\right)}^{2}}}$$

where M denotes the total number of subjects in the test set, ${AHI}_{j}^{pre}$ represents the predicted AHI for the jth subject, and ${AHI}_{j}^{true}$ denotes the corresponding true AHI value for the jth subject. Moreover, ${\bar{AHI}}^{pre}$ and ${\bar{AHI}}^{true}$ represent the mean values of the predicted and true AHI, respectively.

### 4.3. Experimental Results and Ablation Study

**Figure 7:**
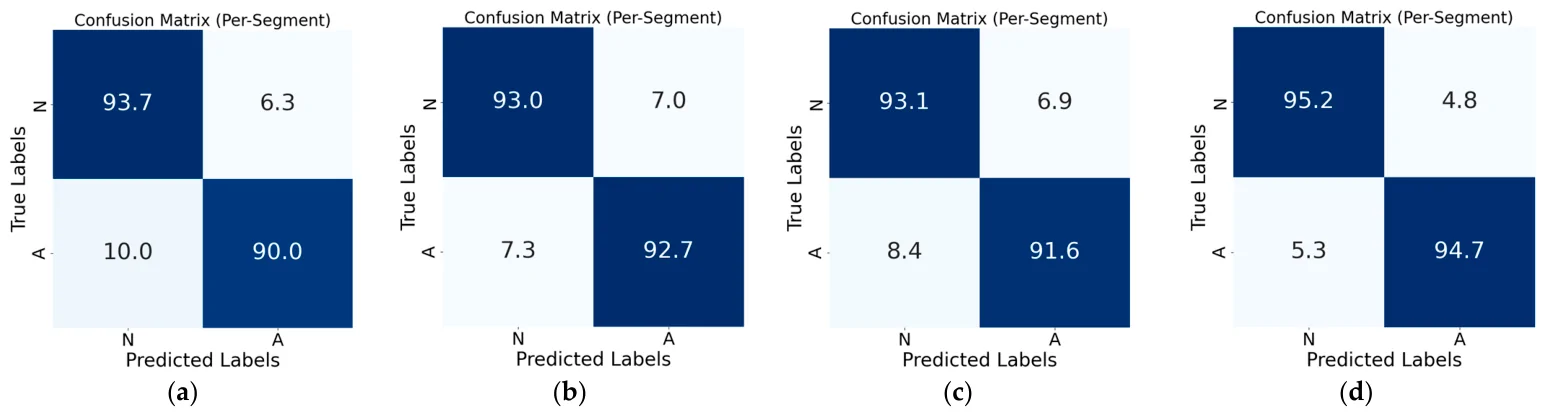
Confusion matrices-normal vs. apneic segments (per-segment). (**a**) Method-1. (**b**) Method-2. (**c**) Method-3. (**d**) SApneaNet.

**Figure 8:**
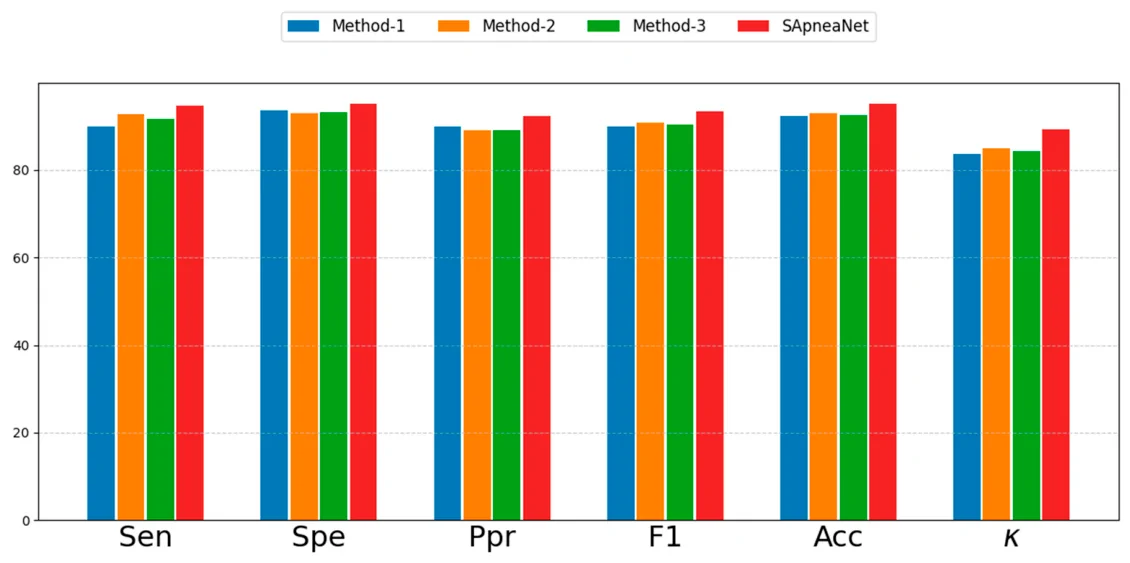
Performance comparison of the methods tested for per-segment SA detection.

**Figure 9:**
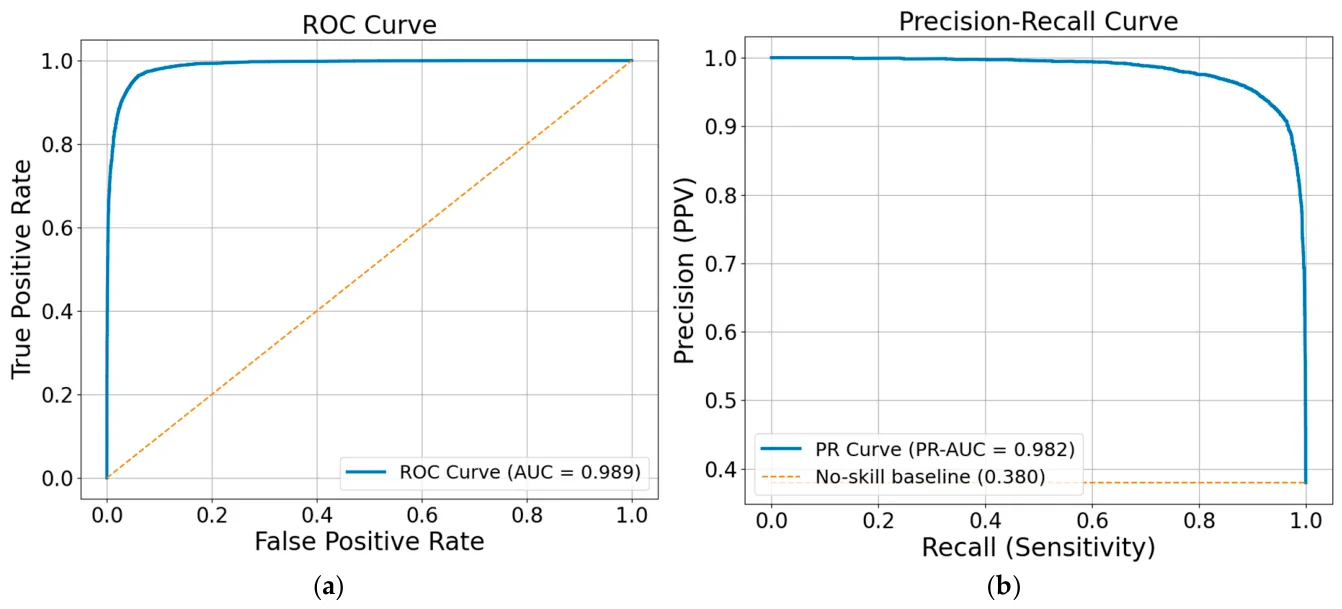
(**a**) ROC curve and (**b**) precision–recall curve of the proposed SApneaNet for segment-level SA detection.

**Figure 10:**
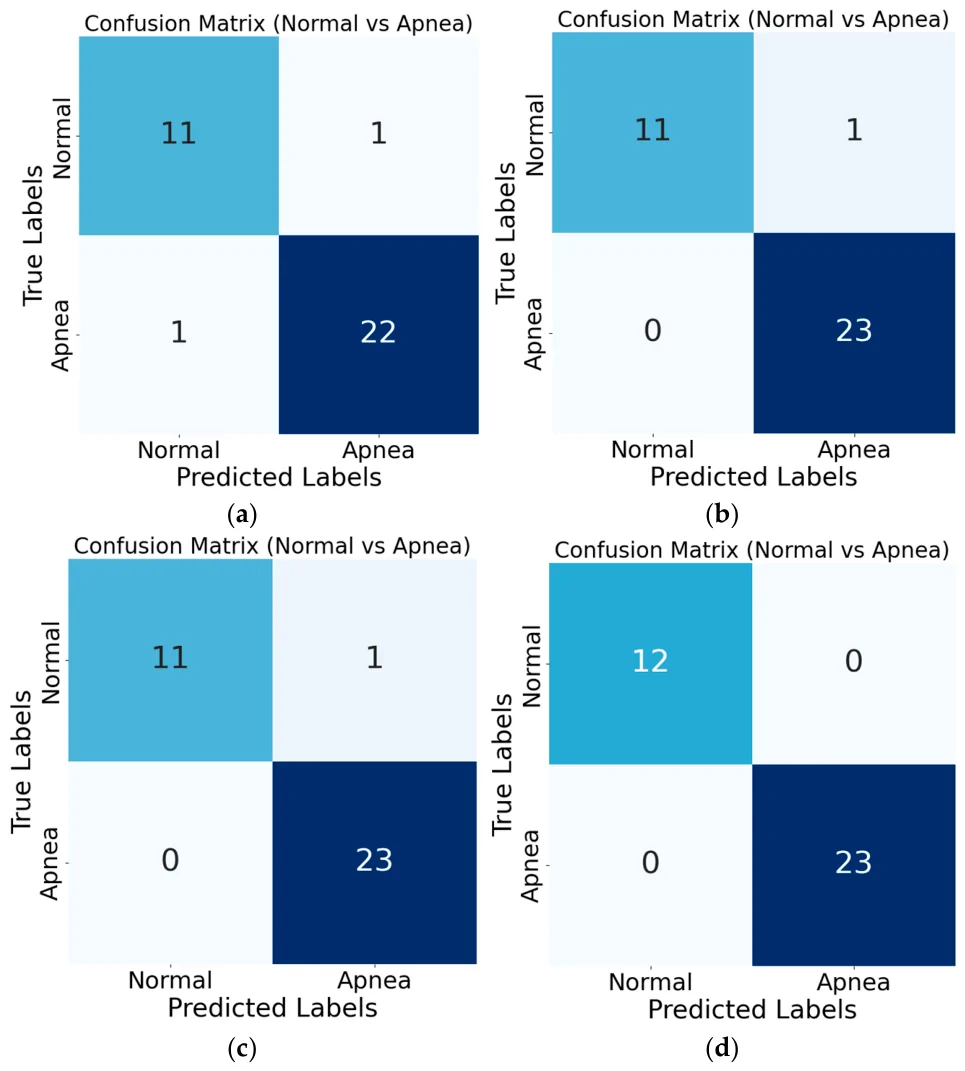
Confusion matrices-normal vs. apneic subjects (per-recording): (**a**) Method-1, (**b**) Method-2, (**c**) Method-3 and (**d**) SApneaNet.

**Figure 11:**
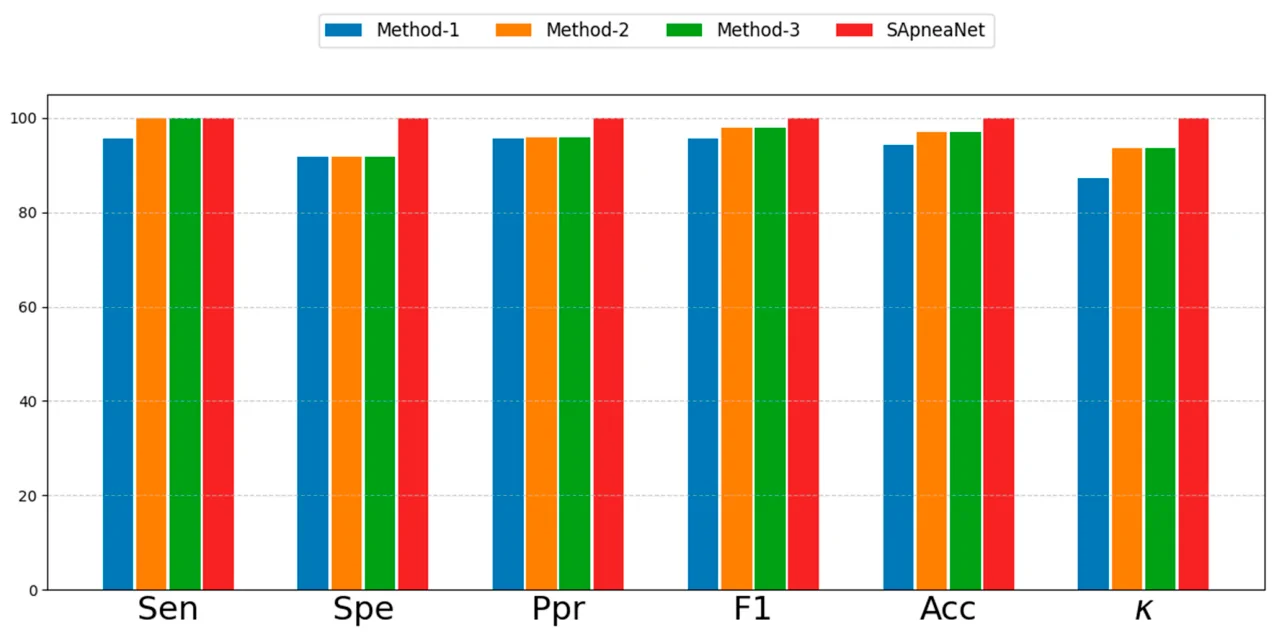
Performance comparison of the methods tested for per-individual SA detection.

**Figure 12:**
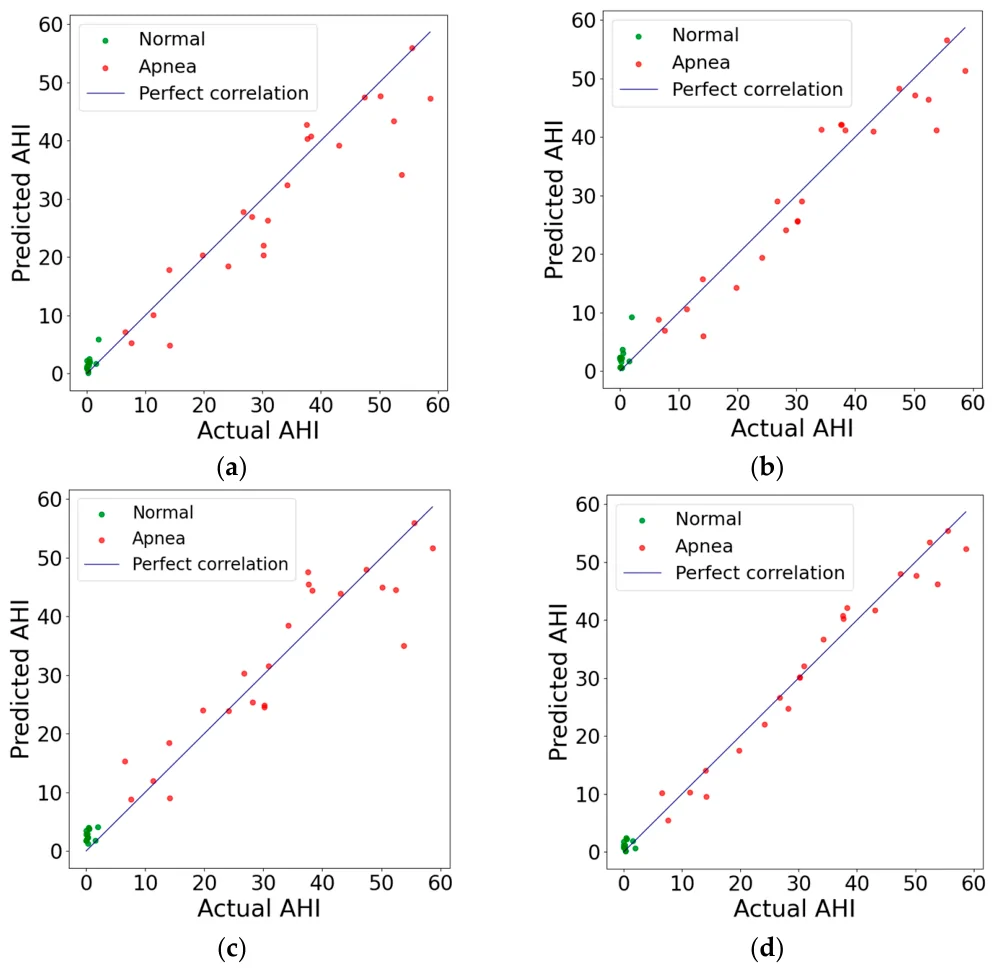
Scatter plots comparing ground-truth and predicted AHI for per-recording evaluation using four models: (**a**) Method-1, (**b**) Method-2, (**c**) Method-3 and (**d**) SApneaNet.

For SA detection, a key objective of the proposed SApneaNet is its ability to accurately identify both apneic segments and affected patients using RGB log-scalogram images. Accordingly, the first step involves evaluating the model’s performance at the segment level, assessing its capability to detect apnea events within individual ECG segments. To validate the effectiveness of the proposed final model and its individual components, we conducted an ablation study on the Apnea-ECG dataset. This study evaluates the contribution and performance of each component by decomposing the final model (see Figure 4) into four configurations: CNN, CNN + ASE, CNN + Transformer, and CNN + ASE + Transformer + AGFF, referred to as Method-1, Method-2, Method-3, and SApneaNet (Method-4), respectively. Each method was assessed for its ability to detect SA under the same hyperparameter settings. The results of the ablation experiments are presented in Table 3 and Table 4, and Figure 7, Figure 8, Figure 9, Figure 10, Figure 11 and Figure 12. The findings indicate that each method, when evaluated independently, demonstrates considerable predictive capabilities.

**Table 3:** Comparison with different model structures on per-segment classification.

Model	Sen	Spe	Ppr	F1	Acc	κ	ROC-AUC	MCC	j
Method-1	90.0	93.7	89.9	89.9	92.3	83.7	0.974	0.837	0.837
Method-2	92.7	93.0	89.1	90.9	92.9	85.0	0.977	0.851	0.857
Method-3	91.6	93.1	89.1	90.4	92.5	84.3	0.976	0.843	0.847
SApneaNet	94.7	95.2	92.3	93.5	95.1	89.4	0.989	0.894	0.899

**Table 4:** Comparison with different model structures on per-recording classification.

Model	Sen	Spe	Ppr	F1	Acc	κ	MAE	PCC
Method-1	95.7	91.7	95.7	95.7	94.3	87.3	3.529	0.968
Method-2	100.0	91.7	95.8	97.9	97.1	93.5	3.359	0.977
Method-3	100.0	91.7	95.8	97.9	97.1	93.5	3.401	0.971
SApneaNet	100.0	100.0	100.0	100.0	100.0	100.0	2.025	0.992

From Table 3 and Figure 8, it can be observed that for per-segment apnea detection, Method-1 achieved a sensitivity of 90.0%, specificity of 93.7%, precision of 89.9%, F1-score of 89.9%, accuracy of 92.3%, and Cohen’s kappa of 83.7%. Method-2 achieved a sensitivity of 92.7%, specificity of 93.0%, precision of 89.1%, F1-score of 90.9%, accuracy of 92.9%, and Cohen’s kappa of 85.0%. Comparing the results of Method-1 and Method-2, it can be observed that sensitivity, F1-score, accuracy, and Cohen’s kappa increased by 2.7%, 1.0%, 0.6%, and 1.3%, respectively. However, slight decreases in specificity and precision are also observed. These results suggest that integrating CNN with the ASE module enhances the model’s ability to capture informative features, particularly improving its sensitivity to apnea events. The ASE module adaptively recalibrates channel-wise feature responses, enabling the network to emphasize salient ECG patterns relevant to OSA detection. This mechanism improves feature discrimination and explains the observed performance gains despite minor trade-offs. Method-3 achieved a sensitivity of 91.6%, specificity of 93.1%, precision of 89.1%, F1-score of 90.4%, accuracy of 92.5%, and Cohen’s kappa of 84.3%. Compared to Method-1, improvements are observed in sensitivity (+1.6%), F1-score (+0.5%), accuracy (+0.2%), and Cohen’s kappa (+0.6%), while slight decreases in specificity and precision are noted. These improvements can be attributed to the transformer encoder’s ability to model global contextual dependencies by operating on tokenized CNN feature maps and applying self-attention, thereby capturing discriminative time-frequency dependencies within the ECG signal representations. This indicates that the global receptive capability of the self-attention mechanism enhances feature extraction by effectively modeling dependencies that are not captured by convolutional operations alone. This complementary behavior improves feature expressiveness beyond what is achieved by CNN alone. When Method-2 is compared with Method-3, Method-2 achieves better overall performance. This indicates that channel-wise feature recalibration through the ASE module is more effective than direct global dependency modeling in this setting. The ASE module enhances CNN-extracted scalogram image features by adaptively emphasizing informative channels, leading to improved feature discrimination. In contrast, the Method-3 architecture relies on tokenized feature representations, which may be less effective when applied directly on scalogram-based CNN features without explicit feature refinement. These results suggest that local feature enhancement through ASE plays a more critical role than standalone global modeling in improving SA detection performance. SApneaNet, which combines CNN, ASE, and transformer with an adaptive gated fusion (AGFF) mechanism, achieved a sensitivity of 94.7%, specificity of 95.2%, precision of 92.3%, F1-score of 93.5%, accuracy of 95.1%, and Cohen’s kappa of 89.4%. Comparing SApneaNet with Method-1, Method-2, and Method-3; SApneaNet demonstrates consistent improvements across all evaluation metrics, confirming its superior robustness and generalization capability of fully exploiting and using the combination of time-frequency image feature information for SA detection. These results demonstrate that the effective integration of CNN, ASE, and transformer (AGFF) enables the model to jointly leverage local feature extraction, channel-wise feature recalibration, and global contextual modeling. In addition, the AGFF module facilitates adaptive feature integration by regulating the contribution of transformer-derived features based on complementary CNN representations. This mechanism enables the model to emphasize more informative global features while suppressing less relevant ones, thereby improving feature discrimination and enhancing the detection of intermittent apnea and hypopnea events in ECG signals. Overall, this synergy significantly enhances the model’s ability to capture discriminative time-frequency characteristics from scalogram images, enabling the extraction of features relevant to SA and thereby improving SA detection performance. Moreover, the effectiveness of the proposed models was investigated using ROC-AUC and PR-AUC, computed for all model variants at the segment level. SApneaNet unequivocally achieved the highest ROC-AUC of 0.989 and PR-AUC of 0.982, indicating excellent discriminative performance, with an ROC-AUC value close to 1 and a favorable precision–recall trade-off despite the class imbalance in the segment-level data. Notably, the PR-AUC substantially exceeds the no-skill baseline of 0.38, corresponding to the proportion of apneic segments in the test set, confirming that this performance is not attributable to class imbalance alone. This suggests a strong capability of the model to distinguish between apnea and normal segments across varying decision thresholds, indicating heightened clinical relevance and considerable potential for clinical application. Furthermore, Figure 9a illustrates the ROC curve of SApneaNet, which remains close to the top-left corner of the plot, reflecting a high true positive rate and a low false positive rate. Figure 9b illustrates the corresponding PR curve, which remains close to the top-right corner, reflecting consistently high precision maintained across a wide range of recall values. These results confirm the superior classification performance and robustness of the proposed SApneaNet for SA detection.

After the per-segment analysis, we evaluated the models’ ability to predict recording-level labels using the AASM diagnostic criteria (AHI ≥ 5). The test set consists of 35 subjects, including 23 apneic and 12 normal individuals. The corresponding classification results are presented in Table 4 and Figure 10 and Figure 11. Method-1 achieved a sensitivity of 95.7%, specificity of 91.7%, precision of 95.7%, F1-score of 95.7%, accuracy of 94.3%, Cohen’s kappa of 87.3%, MAE of 3.529, and PCC of 0.968. Method-2 achieved a sensitivity of 100.0%, specificity of 91.7%, precision of 95.8%, F1-score of 97.9%, accuracy of 97.1%, Cohen’s kappa of 93.5%, MAE of 3.359, and PCC of 0.977. Comparing the two, Method-1 misclassified one apneic subject as normal and one normal subject as apneic, whereas Method-2 misclassified only one normal subject as apneic. This indicates that Method-2 outperforms Method-1; the improvement demonstrates the effectiveness of the ASE module, which enhances feature discrimination by adaptively recalibrating channel-wise feature representations, resulting in the correct detection of all apneic subjects in the test set. This outcome highlights the model’s high sensitivity and its potential clinical relevance in reducing missed apnea cases. Method-3 also misclassified only one normal subject as apneic, and therefore achieved identical classification results to Method-2; however, it falls short in terms of MAE and PCC. The SApneaNet correctly classified all 23 apneic and 12 normal subjects among all methods, achieving perfect per-recording performance with 100% accuracy [95% CI: 90.0–100%, *n* = 35], as well as 100% sensitivity, specificity, precision, F1-score, and Cohen’s kappa. In addition, it obtained the highest PCC (0.992 [95% CI: 0.984–0.996]) and the lowest MAE (2.025), with the per-subject absolute AHI error ranging from 0.000 to 7.510. These results demonstrate the superior effectiveness, exemplary stability and reliability of SApneaNet, consistently outperforming the other models across all evaluation metrics.

As shown in Figure 12 of scatter plots, SApneaNet achieved the highest correlation performance among all models. The predicted AHI values of SApneaNet on the testing set closely match the actual AHI values, as demonstrated in Figure 12d, with a high correlation coefficient of 0.992.

(1) OSA Severity Assessment

**Figure 13:**
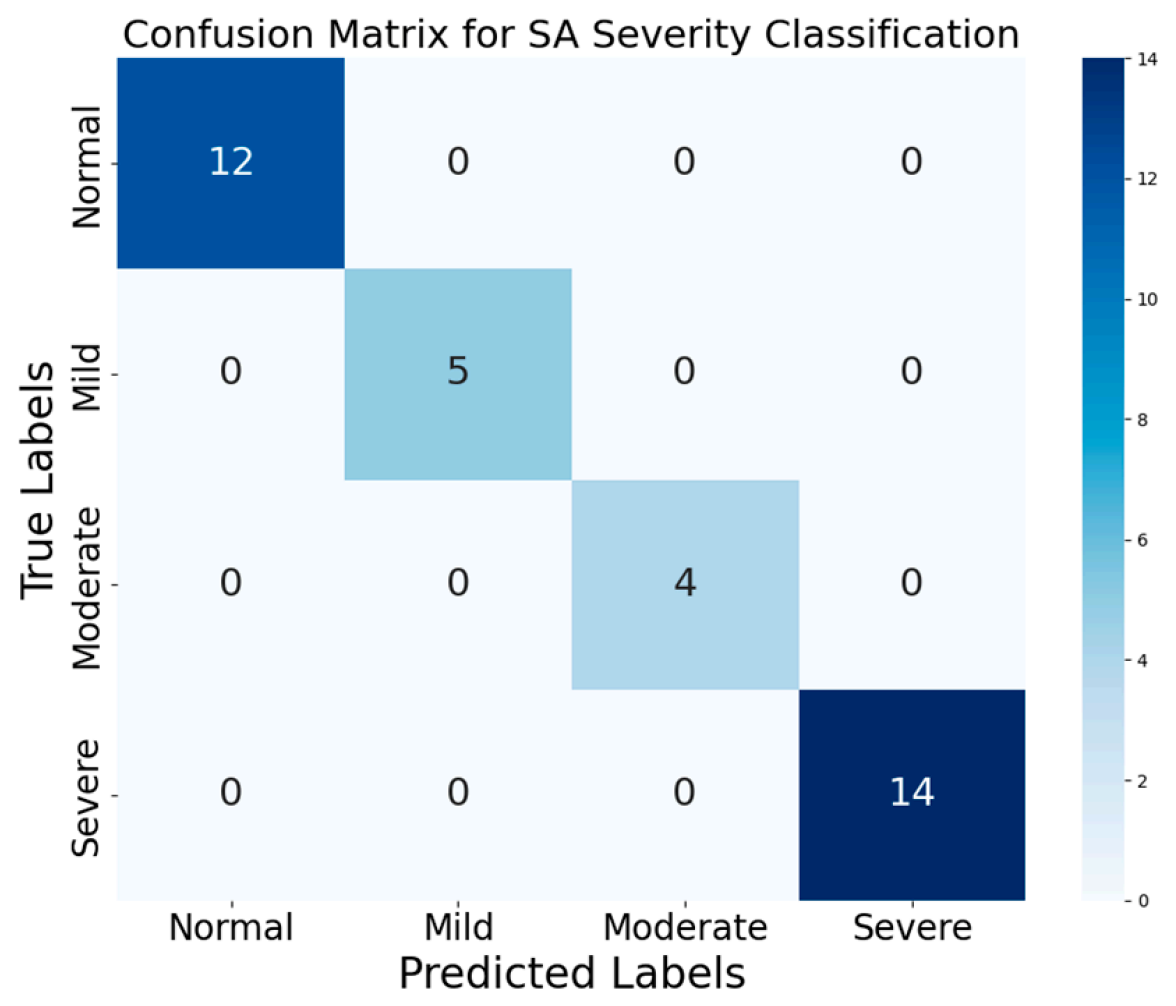
Confusion matrix for individual-level SA severity classification.

To further evaluate the performance of the proposed model and assess its potential in clinical application, SApneaNet was used to classify the severity of OSA at the subject level. Given the importance of individual-level diagnosis in guiding treatment decisions, the proposed model was applied to categorize OSA severity for each subject. Figure 13 presents the confusion matrix of the individual-level diagnostic results. The model achieved a classification accuracy of 100% [95% CI: 90.0–100%, *n* = 35] at this level, demonstrating its capability for precise subject-level diagnosis and severity classification. These results highlight the potential of the proposed method as a supportive tool for clinical assessment of OSA severity.

(2) Visualization of Learned Feature Representations and Discriminative Performance of SApneaNet

**Figure 14:**
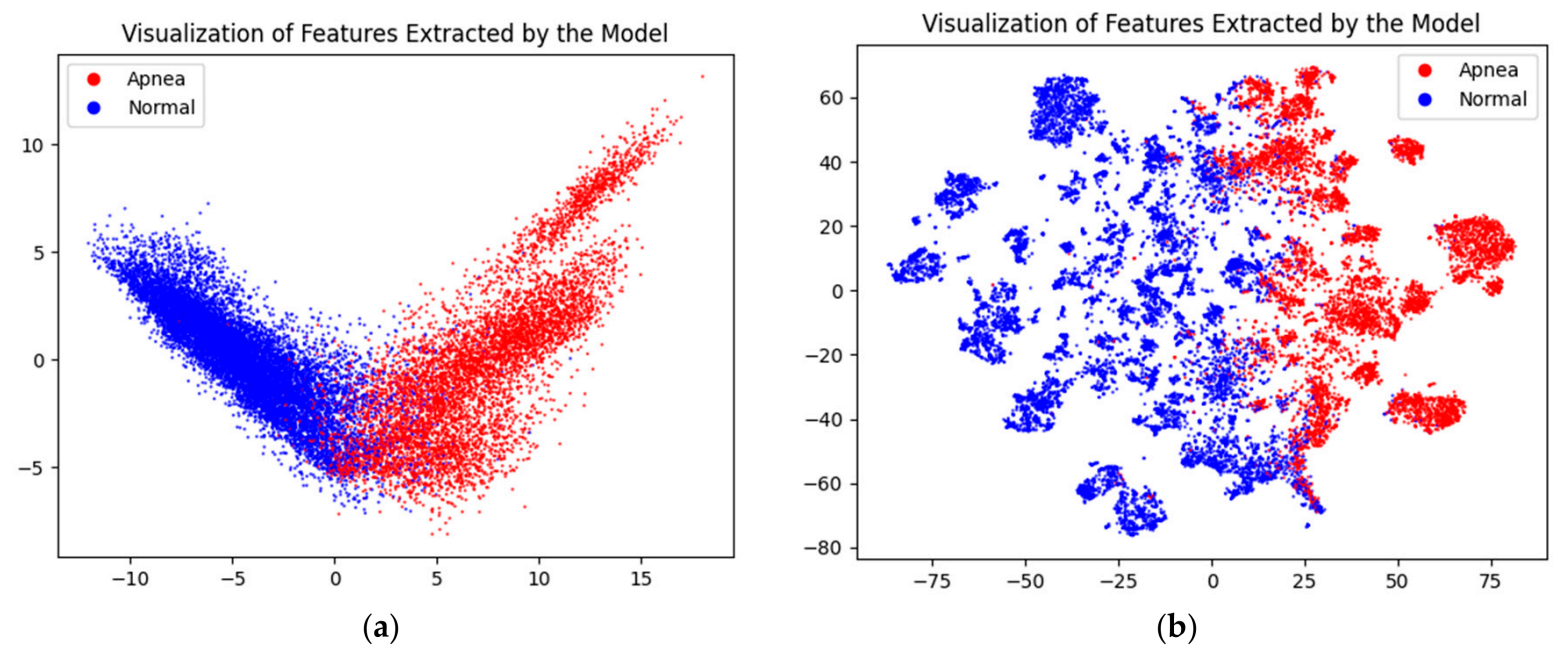
Visualization of feature representations learned by SApneaNet. (**a**) PCA. (**b**) t-SNE-PSO.

To assess the discriminative power of the learned feature representations, features were extracted from the penultimate dense layer and visualized, as shown in Figure 14. The visualization reveals a clear separation between normal and apnea events, with only limited overlap near the decision boundary region. This overlap is mainly observed in transition regions between apnea and normal breathing. This indicates that the proposed SApneaNet model learns highly discriminative feature embeddings that effectively distinguish apnea from normal events. Furthermore, in Figure 14b, the left side shows a scattered cluster of blue points, while the right side shows a scattered cluster of red points, with a small overlap region between the two classes. This pattern suggests that the SApneaNet has learned substructures within each class. Such behavior is common in physiological signals, as both apnea and normal events exhibit inherent intra-class variability and are not perfectly homogeneous.

From the presented results of the proposed SApneaNet, it can be observed that the use of RGB log-scalogram representations provides several advantages for SA detection. By using time-frequency images, the proposed framework captures both temporal and spectral variations associated with respiratory-induced cardiac dynamics. ECG signals are known to be highly correlated with respiratory activity, where apneic events induce characteristic HRV patterns. These variations manifest as unstable and shifting frequency components in the scalogram, in contrast to the relatively stable frequency patterns observed in normal segments as shown in Figure 3. Compared to plain ECG signals, RGB log-scalogram images enhance the visibility of these discriminative frequency variations, enabling clearer observation of apnea-related transitions and facilitating localization of the onset and offset of OSA events. In addition, the time-frequency image allows clinicians to better identify abnormal cardiac dynamics associated with apnea episodes. Furthermore, RGB log-scalogram images enable the model to automatically learn relevant features without manual extraction of R-peak-based characteristics, thereby reducing dependency on expert-driven feature engineering.

Building upon these representational advantages, the proposed SApneaNet effectively captures discriminative cardiac patterns associated with SA. By transforming ECG signals into time-frequency images, the model learns feature representations that emphasize low- to mid-frequency components linked to heart rate variability changes occurring during apneic events. These include rhythm disturbances such as bradycardia, tachycardia, and atrial rhythm irregularities, as well as ST-segment and QT-related abnormalities, which are clinically reported to be associated with conditions such as myocardial ischemia. The CNN and ASE blocks enhance local feature refinement in scalogram images, while the transformer with gated fusion captures higher-level contextual dependencies. This combination enables robust discrimination between stable non-apneic rhythms and pathological variations induced by oxygen desaturation during apnea episodes.

## 5. Comparison and Discussion

Most previous studies rely on manually engineered physiological representations [4,21,22,24,32,34] and handcrafted feature extraction [31,40,41,42,43,44,45] from ECG signals for classification. However, such feature engineering procedures are often tedious, labor-intensive, time-consuming, and require substantial domain expertise. Feature computation and engineering are often computationally intensive stages in traditional ML pipelines, particularly when applied to high-dimensional representations. Their performance heavily depends on the compatibility between the extracted feature set and the selected classifier, while inaccuracies during feature extraction may further degrade SA classification performance. Moreover, recent regular DL models often exhibit limited generalization ability, resulting in suboptimal SA prediction performance. Therefore, the proposed model effectively addresses these limitations. Thus, the direct use of RGB log-scalogram representations enables automatic feature learning, providing a more efficient and robust approach for SA detection, while minimizing manual intervention, eliminating reliance on handcrafted feature extraction, significantly reducing human effort, and decreasing dependence on prior expert knowledge. In addition, RGB log-scalogram enhances the performance of SA detection at the same time. The proposed SApneaNet integrates CNN, ASE, transformer, and AGFF components to address data hunger and enhance the detection of subtle apnea-related ECG patterns, leading to improved SA prediction performance.

To the best of our knowledge, this is the first study to employ RGB log-scalogram image with a CNN + ASE + Transformer (AGFF) architecture for SA detection. Our proposed model, being a new framework, requires comprehensive evaluation and comparison with germane recent SOTA methods to assess its effectiveness and reliability. Table 5 and Table 6 present the comparative results for both per-segment and per-recording SA detection. To ensure a fair and meaningful comparison, only studies evaluated on the same Apnea-ECG database were considered. The comparison results demonstrate that our novel proposed method achieves competitive performance and effectiveness relative to existing approaches.

**Table 5:** Performance comparison with previous methods for per-segment SA detection on the PhysioNet Apnea-ECG dataset.

Study	Year	Input Type	Method	Acc	Sen	Spe	F1	κ	ROC-AUC
[41]	2016	RRI, EDR	HMM + SVM	86.2	82.6	88.4	-	-	0.940
[44]	2017	TQWT	RUSBoost	88.9	87.6	91.5	-	-	-
[33]	2018	RRI	DNN + HMM	84.7	88.9	82.1	-	-	0.869
[28]	2019	CWT	AlexNet CNN	86.2	90.0	83.8			0.881
[43]	2020	HR, RR Signals	FAEMD + SVM	80.5	82.3	78.7	81.0	80.0	0.610
[32]	2021	RRI	FSSAE + HMM	85.1	86.2	84.4	-	-	-
[24]	2021	RA, RRI	CNN + LSTM + SVM	90.9	91.2	90.3	92.7	-	-
[40]	2021	DWT	CFS + PSO + RF	90.3	86.6	92.6	87.3	-	0.960
[31]	2022	HRV Features + 2D Image	BiLSTM + SqueezeNet	91.5	91.0	91.9	-	-	-
[27]	2022	CWT + GADF	CNN + Voting mechanism	90.9	83.7	95.3	-	-	0.890
[4]	2022	Raw ECG, RA, RRI, RRID	CNN + Transformer	90.5	86.5	93.0	87.5	-	0.961
[50]	2022	EMD	SMOTE + CNN	91.8	91.0	92.6	-	-	0.973
[21]	2023	RA, RRI	CNN + Transformer	91.7	89.2	93.3	89.1	-	-
[25]	2023	Raw ECG	CNN + Transformer	88.2	78.5	94.1	89.0	-	0.947
[29]	2024	Raw ECG	MLCNN + RFEFS + OBiLSTM	92.9	-	-	-	-	-
[34]	2024	RA, RRI	CNN + Transformer	92.1	88.4	94.4	89.5	-	-
[26]	2025	CWT + LIME	GoogLeNet CNN	93.9	93.4	94.3	93.8	-	-
[30]	2025	Raw ECG	CNN + BiLSTM	88.6	84.2	91.0	84.9	75.7	0.940
[22]	2025	EDR, RRI, RAMP	CNN + Transformer	95.0	93.3	-	-	-	0.969
Ours	2026	RGB Log-Scalogram	SApneaNet	95.1	94.7	95.2	93.5	89.4	0.989

**Table 6:** Performance comparison with previous methods for per-recording SA detection on the PhysioNet Apnea-ECG dataset.

Study	Year	Input Type	Method	Acc	Sen	Spe	Ppr	F1	MAE	PCC
[41]	2016	RRI, EDR	HMM + SVM	97.1	95.8	100	-	-	-	-
[33]	2018	RRI	DNN + HMM	100	100	100	-	-	-	-
[28]	2019	CWT	AlexNet	100	100	100	-	-	-	-
[9]	2021	CWT + EMD	CNN	100	100	100	-	-	-	-
[32]	2021	RRI	FSSAE + HMM	97.1	95.7	100	-	-	5.60	-
[4]	2022	Raw ECG, RA, RRI, RRID	CNN + Transformer	100	100	100	100	100	2.71	-
[31]	2022	HRV Features + 2D Image	BiLSTM + SqueezeNet	100	100	100	-	-	-	-
[25]	2023	Raw ECG	CNN + Transformer	100	-	-	-	-	4.33	-
[21]	2023	RA, RRI	CNN + Transformer	97.1	95.7	100	-	-	2.45	-
[23]	2024	STFT Spectrogram	EfficientNet B0 + SVM	97.1	100	91.7	-	-	-	-
[30]	2025	Raw ECG	CNN + BiLSTM	94.3	100	83.3	-	-	-	0.969
[22]	2025	EDR, RRI, RAMP	CNN + Transformer	100	100	100	100	100	-	-
Ours	2026	RGB Log-Scalogram	SApneaNet	100	100	100	100	100	2.02	0.992

As shown in Table 5, other existing studies have also achieved good per-segment performance. In terms of per-segment accuracy, SOTA methods lagged behind the proposed SApneaNet, which achieved a 0.1% improvement over the best-performing model among the comparative studies [22]. This demonstrates the effectiveness of the proposed framework for SA detection and highlights its ability to accurately classify SA segments. The high classification performance is attributed to the integration of CNN, ASE, and transformer modules with the AGFF mechanism, which collectively enhance feature extraction and contextual representation learning. Thus, the proposed model effectively distinguishes between SA and non-SA events, contributing to reliable overall detection performance among the compared SOTA approaches. Moreover, our model attains a higher sensitivity with an increase of 1.3%, compared with the best-performing SOTA model [26]. This improvement indicates a stronger capability in correctly identifying apneic events, which is a critical factor in clinical settings. High sensitivity is particularly important in practical applications, as it minimizes missed apnea events and supports timely clinical intervention. Furthermore, although our model maintains a competitive specificity of 95.2%, the method reported in [27] achieved a slightly higher specificity of 95.3%, with only a marginal difference of 0.1%. However, their method [27] exhibits a substantially lower sensitivity of 83.7%, indicating reduced capability in detecting apnea events and revealing an imbalance between sensitivity and specificity that may suggest bias toward the negative class. In contrast, our proposed model provides a more balanced trade-off between specificity (95.2%) and sensitivity (94.7%), providing reliable detection performance while minimizing both false-positive and false-negative predictions. Our F1 score is among the highest reported, where the method reported in [26] achieved a slightly higher F1-score of 93.8%, compared with 93.5% obtained by our proposed model, the difference is only marginal (0.3%). Moreover, its [26] accuracy, sensitivity, and specificity are lower than those achieved by our SApneaNet by 1.2%, 1.3%, and 0.9%, respectively. Several previous studies did not report Cohen’s kappa and ROC-AUC values, making comprehensive evaluation of classification consistency and discriminative capability difficult. In contrast, our proposed model achieved the highest reported Cohen’s kappa and ROC-AUC values of 89.4% and 0.989, respectively, demonstrating strong agreement between predicted and true labels as well as excellent classification reliability. Furthermore, the model maintains a balanced performance across all evaluation metrics, achieving high sensitivity for accurate apnea event detection while preserving high specificity to reduce false-positive predictions. These results highlight the robustness, stability, practical suitability, exceptional predictive power, and strong generalization ability of the proposed framework for real-world SA detection applications.

Table 6 presents the per-individual SA detection results. The proposed SApneaNet achieves highly competitive performance and perfectly distinguishes between SA and normal subjects. Notably, five studies [21,23,30,32,41] did not attain perfect classification performance of 100% accuracy. Similarly, we observed that most existing studies did not report the MAE and PCC metrics for per-recording evaluation, despite their importance in assessing OSA severity. Although Chenguang Li et al. reported a strong MAE of 2.45 in their study [21], our proposed method achieved a lower MAE of 2.02, indicating its superior capability for OSA severity grading evaluation, which is of considerable clinical importance for the application of the proposed model in practical scenarios. Furthermore, their method [21] misclassified one apneic subject, which could potentially delay appropriate clinical treatment attention and prevented their model from achieving 100% perfect classification performance. In terms of PCC, although Hossan et al. reported a strong PCC of 0.969 in their study [30], our proposed method achieved a higher PCC of 0.992, further demonstrating its advantages and effectiveness for OSA severity assessment. Moreover, their method [30] misclassified two normal subjects, which may lead to unnecessary clinical concern or further examination, preventing their model from achieving 100% perfect classification performance. In addition, many existing studies focused only on differentiating apneic subjects from normal subjects, while relatively few investigated OSA severity assessment classification into mild, moderate, and severe categories. The study reported in [4] performed severity assessment and achieved an accuracy of 82.6%, where one mild case was misclassified as moderate, one moderate case as severe, and two severe cases as moderate. In contrast, as shown in Figure 13, our proposed method achieved 100% severity classification accuracy, demonstrating its strong capability to capture discriminative ECG patterns associated with SA events. These results further confirm the model’s exceptional predictive power and strong generalization ability among the compared SOTA approaches.

### 5.1. Computational Efficiency and Broader Applicability of SApneaNet

The proposed method achieves highly competitive performance, making it efficient, cost-effective, and suitable for practical real-world clinical applications, with strong potential for broader deployment in scenarios requiring reliable and precise SA detection. To support this claim, we evaluate the computational efficiency and storage requirements of the SApneaNet. All computational measurements were obtained on a computer equipped with an Intel^®^ Core™ i9-10900 CPU @ 2.80 GHz and 32 GB of RAM (Santa Clara, CA, USA). In our setup, storing a processed 9 h17 min ECG recording requires approximately 25 MB, with an average processing time of 0.06461 s per ECG segment. In addition, the model requires approximately 2.38 MB of storage, while achieving an inference time of 0.03351 s per RGB log-scalogram. Thus, following lightweight model optimization with guaranteed model detection accuracy, the SApneaNet holds the potential to be integrated into wearable medical devices, enabling early SA detection, timely intervention, and treatment, thereby improving sleep quality for individuals affected by the disorder. Beyond SA detection, the proposed model, due to its strong feature extraction capability, can be readily adapted to other biomedical and signal processing tasks, such as cardiovascular disease analysis and arrhythmia classification, by appropriately adjusting the input data, channels, output classes, and network configuration according to the target application.

### 5.2. Future Research Directions

Although the proposed method achieves strong performance, its practical application in clinical SA diagnosis still faces certain challenges due to the inherent complexity of the SA diagnosis problem. First, the dataset used in this study is an open-access competition dataset with a relatively moderate size and an imbalanced sex distribution. As a result, the advantages of DL models, which typically require large-scale data for optimal performance, cannot be fully exploited. Second, the proposed model has not yet been applied on real-world clinical hospital data. Therefore, we are addressing this challenge through active collaboration with senior residences, medical centers, and hospitals to collect a larger and more diverse patient dataset, including variations in health conditions, demographic factors, and clinical settings. This expanded database will help improve model robustness and performance, while also supporting clinicians in more accurate SA labeling and diagnosis.

Beyond these data-related challenges, broader extensions of the proposed framework are also envisioned. Our model holds the potential to be extended to remote SA diagnosis within the internet of medical things (IoMT). In future work, upon the collection and integration of medical data from additional sensor modalities such as EEG, EMG, PPG, SpO_2_, and airflow signals, further rigorous experiments will be conducted using the proposed model to improve its ability to recognize other different types of SA, including CSA and MSA, thereby providing insights relevant to clinical practice. As mentioned, our model demonstrated the potential for real-time SA detection and integration into IoMT devices. Therefore, a cloud-based IoT system could enable real-time SA detection by securely collecting ECG data, converting it into RGB log-scalograms, and processing them using our DL model for continuous analysis. Such a system would require a reliable and robust infrastructure to support real-time apnea monitoring and timely clinical intervention. However, several challenges would need to be addressed, including preprocessing latency, computational efficiency, system stability, and integration with clinical workflows.

## 6. Conclusions

In this study, we propose a novel OSA detection framework based on single-lead ECG signals, in which ECG signals are transformed into time-frequency RGB log-scalogram representations and subsequently fed into the proposed SApneaNet architecture to capture discriminative spectral, temporal, and physiological characteristics associated with SA events for accurate apnea detection. Notably, unlike conventional and regular CNN–Transformer architectures, the proposed CNN + ASE + Transformer (AGFF) framework jointly extracts rich local features, performs channel-wise feature recalibration, and captures global contextual dependencies. The ASE module enhances feature refinement by suppressing redundant information while emphasizing informative representations, whereas AGFF enables adaptive fusion of CNN and transformer features. This paradigm eliminates manual feature extraction, thereby reducing reliance on expert knowledge and minimizing potential sources of error. Experimental results on the PhysioNet Apnea-ECG dataset demonstrate that the proposed framework achieves excellent and competitive performance compared with SOTA methods, with a sensitivity of 94.7%, specificity of 95.2%, precision of 92.3%, F1-score of 93.5%, accuracy of 95.1%, Cohen’s kappa of 89.4%, and an ROC-AUC of 0.989 for per-segment classification, thereby significantly improving OSA detection performance. Furthermore, per-recording evaluation confirms reliable OSA severity assessment with the lowest MAE (2.025) and highest PCC (0.992), while per-subject classification achieves 100% accuracy, suggesting that the proposed method closely aligns with expert-like diagnostic behavior. After lightweight optimization, the proposed model can be integrated into wearable and ECG-based systems for real-time SA detection in both clinical and home care settings, supporting early diagnosis, timely intervention, and improved sleep quality, and ultimately contributing to better patient outcomes.

## Figures and Tables

**Table 1:** Description of the PhysioNet Apnea-ECG Dataset.

Dataset	Training Set	Test Set	Total	%
Apnea	6514	6552	13,066	38
Normal	10,611	10,751	21,362	62
Total	17,125	17,303	34,428	100
%	49.7	50.3	100	
No. of Patients	35	35	70	
Average Age	46.5	43.8		
Average BMI	28	28		

**Table 2:** Parameters of CNN.

Layer	Filters	Kernel Size	Output Dimension
Input			64 × 64 × 3
Conv2D1 + BN1 + ReLU1	64	3 × 3	64 × 64 × 64
MaxPool2D1 (2×2)	NA	2 × 2	32 × 32 × 64
Conv2D2 + BN2 + ReLU2	128	3 × 3	32 × 32 × 128
MaxPool2D2 (2×2)	NA	2 × 2	16 × 16 × 128
ASE	NA	NA	16 × 16 × 128
Conv2D3 + BN3 + ReLU3	256	3 × 3	16 × 16 × 256
MaxPool2D3 (2×2)	NA	2 × 2	8 × 8 × 256

## Data Availability

This study used the PhysioNet Apnea-ECG Database, originally provided by Philipps-University, Marburg, Germany [46], and accessed through PhysioNet [47] at https://www.physionet.org/content/apnea-ecg/1.0.0/ (accessed on 08 October 2025).
